# CAMKK2-CAMK4 signaling regulates transferrin trafficking, turnover, and iron homeostasis

**DOI:** 10.1186/s12964-020-00575-0

**Published:** 2020-05-27

**Authors:** Mohammad Golam Sabbir

**Affiliations:** 1 Canadian Centre for Agri-Food Research in Health and Medicine, St. Boniface Albrechtsen Research Centre, Room R2034 - 351 Taché Avenue, Winnipeg, MB R2H 2A6 Canada; 2 Alzo Biosciences Inc., San Diego, CA USA

**Keywords:** CAMKK2, CAMK4, transferrin, transferrin-receptor, trafficking, iron homeostasis, glycolysis, respiration, cerebellum, liver

## Abstract

**Background:**

Circulatory iron is a hazardous biometal. Therefore, iron is transported in a redox-safe state by a serum glycoprotein - transferrin (**TF**). Different organs acquire iron from the systemic circulation through a tightly regulated mechanism at the blood-tissue interface which involves receptor-mediated internalization of TF. Thus, abnormal TF trafficking may lead to iron dyshomeostasis associated with several diseases including neurodegeneration. Iron -induced toxicity can cause neuronal damage to iron-sensitive brain regions. Recently, it was discovered that CAMKK2, a calcium (Ca^2+^)/calmodulin-activated kinase, controls receptor-mediated TF trafficking in mouse tissues, specifically in the brain. The biological function of CAMKK2 is mediated through multiple downstream effectors. Both CAMKK2 and one of its downstream kinase, CAMK4, exhibit overlapping expression in mouse brain. The role of CAMK4 in vesicular transport has been reported and loss of CAMKK2 or CAMK4 leads to cognitive defects in mouse. Therefore, it was hypothesized that CAMKK2-CAMK4 signaling regulates receptor-mediated TF trafficking and iron homeostasis which may be responsible for the neuronal malfunction observed in CAMKK2- or CAMK4-deficient mice.

**Methods:**

CAMK4^−/−^ mouse was used to study tissue-specific turnover of TF, TF-receptor (**TFRC**) and iron. CRISPR/Cas9-based CAMKK2 and/or CAMK4 deleted human embryonic kidney-derived HEK293 cell clones were used to study the molecular defects in receptor-mediated TF trafficking. Further, a “zero functional G protein” condition in HEK293 cell was exploited to study CAMKK2-CAMK4 signaling-mediated regulation of intracellular Ca^2+^ homeostasis which was linked to calcium signaling during TF trafficking.

**Results:**

Loss of CAMK4 leads to abnormal post-translational modifications (**PTMs**) and turnover of TF in mouse cerebellum and liver which was associated with iron dyshomeostasis in these tissues. The HEK293 cell-based study revealed that the absence of CAMKK2-CAMK4 signaling altered intracellular Ca^2+^ homeostasis and lead to abnormal calcium signaling during TF trafficking. Also, CAMKK2-CAMK4 signaling deficiency affected the molecular interaction of TF and TF-receptor-associated protein complexes which indicated a potential failure in the recruitment of interacting proteins due to differential PTMs in TF.

**Conclusion:**

Overall, this study established a novel mechanistic link between intracellular Ca^2+^ level, receptor-mediated TF trafficking, and iron homeostasis, all regulated by CAMKK2-CAMK4 signaling.

Video Abstract

**Graphical abstract:**

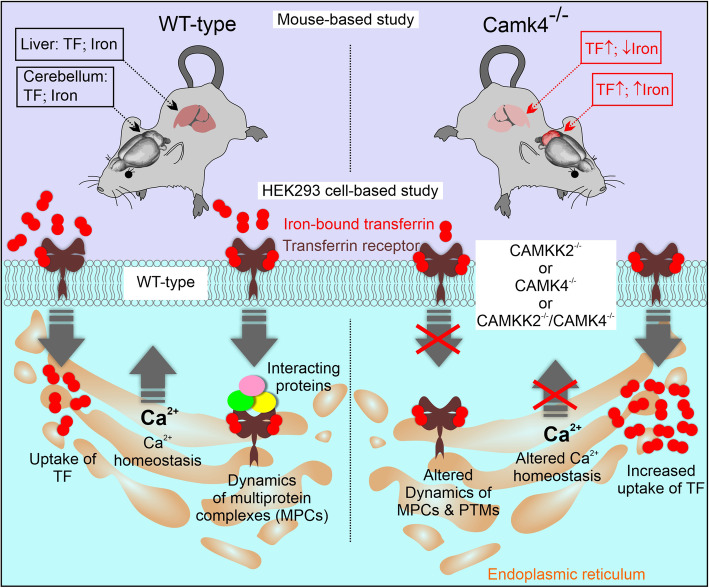

## Background

Iron is an integral part of the haem and iron-sulfur (Fe-S) cluster, and acts as a co-factor for numerous key enzymes involved in metabolic reactions [1]. However, free iron can promote the free radical formation, resulting in oxidative damage [2]. Therefore, iron is transported in a redox-inactive safe-state by transferrin (**TF**), an iron-transporter serum glycoprotein. Under physiological conditions, almost all iron in the circulation is bound to TF [3, 4]. Circulating TF, secreted by the liver harvests iron from the intestinal luminal epithelial cells and delivers it to different organs [5]. Iron-saturated TF binds to the transferrin receptor (**TFRC**) at the luminal surface of the cell and transported inside the cell by endocytosis [6]. Once inside the cell, the endosomal TF may release its cargo and is recycled back to the luminal surface or it may undergo endosomal transcytosis and released to the basal cell-surface [6]. Thus, any disruption to receptor-mediated TF trafficking will lead to iron dyshomeostasis which may interfere with cell function. For example, a hypotransferrinemic mouse that harbors a splicing defect in Tf gene resulting in minimal serum Tf, exhibits extensive deposits of iron in different organs [7, 8]. Therefore, it is important to study the regulatory mechanism controlling receptor-mediated TF trafficking to understand disease pathologies associated with iron dyshomeostasis.

While receptor-mediated TF trafficking has been extensively studied, regulation of this pathway by protein-kinases was not well known until recently. It has now been reported that Ca^2+^/Calmodulin (**CAM**)-Dependent Protein Kinase Kinase-2 (**CAMKK2**) controls phosphorylation and trafficking of TF in vitro and in vivo [9]. Loss of Camkk2 in mice lead to tissue-specific aberrant turnover of Tf [9]. Also, in vitro studies using CRISPR/Cas9-based CAMKK2 knockout (**KO**) HEK293 (human embryonic kidney-derived) and HepG2 (hepatoma-derived) cells showed that CAMKK2 loss interfered with TF trafficking and turnover [9]. CAMKK2 is a serine/threonine (Ser/Thr) kinase that is activated by an increase in the intracellular calcium ion (**Ca**^**2+**^) concentration (**[Ca**^**2+**^**]**_**i**_) and subsequent CAM binding [10]. Therefore, it is conceivable that any disruption in intracellular Ca^2+^ may interfere with CAMKK2 function leading to abnormal TF trafficking which in turn may cause iron dyshomeostasis and affect cell function. Multiple signaling pathways associated with neurodegenerative diseases regulate CAMKK2 function either through intracellular Ca^2+^ release or through glycogen synthase kinase 3 (**GSK3**)/ cyclin-dependent kinase 5 (**CDK5**)/ cyclic AMP (**cAMP**)-activated protein kinase A (**PKA**)-mediated control of CAMKK2 activity. For example, cholinergic signaling can activate CAMKK2 through intracellular Ca^2+^ release [11–19]. Alternatively, CAMKK2 upstream kinases, CDK5, GSK3, and PKA, control CAMKK2 activity by specific activating or inactivating phosphorylation events [20, 21]. CDK5 is a neuron-specific kinase that has been linked to an array of neurodegenerative disorders [18, 19]. GSK3 has been implicated in neurodegenerative diseases [12–17]. Emerging evidence suggests that disruption of intracellular Ca^2+^ homeostasis plays an important role in orchestrating pathogenesis of the degenerative brain disease, associated memory loss and cognitive dysfunction [22]. Therefore, altered Ca^2+^ in neurodegenerative diseases may affect CAMKK2 function leading to increased TF turnover and iron deposition in the brain which may lead to iron-induced toxicity and neuronal damage.

Like calcium, iron is another important bio-metal responsible for neuronal damage to iron-sensitive brain regions [23]. In the aging brain, iron content gradually increases [24], but in neurodegenerative diseases, specifically Alzheimer’s disease (**AD**), brain iron content showed a dramatic increase [25]. The link between disrupted intracellular Ca^2+^ signaling and iron-dyshomeostasis mediated neurodegeneration was not well established until recently when it was reported that abnormal Camkk2 in a triple-transgenic mouse model of AD (3xTg-AD) is associated with altered TF phosphorylation and TF-associated protein complexes in different regions of the brain [9]. Also, whole body Camkk2 deleted mice presented with neurological abnormalities including abnormal spatial learning, impaired long term memory and abnormal long term potentiation at the CA1 synapse, a sign of cognitive malfunction [26]. Based on these evidences, a link between abnormal Ca^2+^ signaling and iron dyshomeostasis-mediated neurodegeneration can be suggested due to aberrant CAMKK2 signaling. Therefore, it is important to study the effects of CAMKK2 downstream effectors on receptor-mediated TF trafficking and calcium/iron homeostasis.

Active CAMKK2 phosphorylates and activates three major downstream kinases: Ca^2+^/CAM Dependent Protein Kinase I (**CAMK1**), Ca^2+^/CAM Dependent Protein Kinase IV (**CAMK4**) and AMP-activated Protein Kinase (**AMPK**) [27]. This leads to the regulation of cell growth as observed in neurite elongation and branching [28], cell cycle control [29], energy balance [30–32], gene expression and protein synthesis [33, 34]. Indirect evidence suggests that CAMK4 may be the potential kinase involved in CAMKK2 downstream signaling-mediated TF trafficking. This prediction is based on a genome-wide analysis of the human kinases involved in caveolae-mediated endocytosis of infectious virus particles, which revealed that silencing of CAMK4 in HeLa cells leads to disturbing vesicular structures containing fluorescent-labeled caveolin in a simian virus 40 (SV40) infectious particle entry assay [35]. This indicates that CAMK4 is involved in vesicular transport and, therefore, may be involved in the vesicular trafficking of TF as well. Also, Tf, Tfrc, Camkk2, and Camk4, have overlapping expression patterns in different regions of mouse brain, specifically in the cerebellum. This was revealed by Tf-promoter-trapped reporter expression and in situ hybridization (**ISH**)-based studies involving Camkk2, Camk4, and Tfrc as documented in the Gene Expression Nervous System Atlas (**GENSAT**) [36] and Brain Gene Expression Map (**BGEM**) projects [37]. Taking these findings together, it was hypothesized that CAMKK2-CAMK4 signaling regulates receptor-mediated TF trafficking and iron homeostasis. In this study, Camk4^−/−^ mouse as well as, multiple CRISPR/Cas9-based CAMKK2, CAMK4, and CAMKK2 + CAMK4 deleted HEK293 cell clones were used to study CAMKK2-CAMK4 signaling-mediated TF trafficking and iron homeostasis. Also, a “zero functional G” HEK293 cell line created by genetic ablation of GαS/L/Q/11/12/13 genes [38] was used to assess the role of CAMKK2-CAMK4 signaling in the maintenance of intracellular Ca^2+^ homeostasis during TF trafficking.

## Methods

### Camkk2^−/−^ and Camk4^−/−^ mouse tissues

The 3–4 months old Camkk2^−/−^, Camk4^−/−^ and wild-type male C57BL/6 J mice brain and liver tissues were provided as dissected snap-frozen tissues by Dr. Uma Sankar, Indiana University School of Medicine, USA. The Camkk2^−/−^ mouse was generated by targeted deletion of exons 2–4 flanking sequence [30]. The Camk4^−/−^ mouse was generated by targeted deletion of first two exons including two known transcription initiation sites of Camk4 [39].

### Cell culture

The CAMKK2^−/−^ HEK293 cells were generated as reported previously [9]. The CRISPR/Cas9 based ΔGsix_0_ HEK293 cells were obtained from the laboratory of Dr. Asuka Inoue, Tohoku University, Japan [38]. The CAMK4^−/−^ and CAMKK2^−/−^/CAMK4^−/−^ (double knockout: **DKO**) cell clones were generated by co-transfecting HEK293 with CAMK4/CAMKK2 CRISPR/Cas9 knockout plasmids (Table-1) and CAMK4/CAMKK2 specific homology-directed repair (HDR) plasmids (Table-1) respectively using lipofectamine 3000 reagent (Catalog: L3000001; ThermoFisher Scientific). The targeted cells were sorted using GFP/RFP-reporter expression (Fig. S1A-C) and subsequently, puromycin resistant clones were selected and screened by immunoblotting using anti-CAMK4 and anti-CAMKK2 antibodies (Fig. S1D-H). The floxed reporter/selection cassette was subsequently removed by transient expression of Cre-recombinase. All cell lines were cultivated in Dulbecco’s modified Eagle’s medium (DMEM) supplemented with 10% heat-inactivated FBS and 1X antibiotic antimycotic solution (Catalog: A5955; Sigma).

### Plasmids and transfection

The RFP-TFRC plasmid (Catalog: plasmid#6105; Addgene) was obtained through the Addgene plasmid repository. The FLAG-TF was obtained from GenScript (Catalog: OHu26129; GenScript). The plasmids were transfected using Lipofectamine 3000 reagent.

### RNA extraction, cDNA synthesis, reverse-transcription PCR (RT-PCR) and DNA sequencing

Total RNA from a C57BL/6 J mouse-derived tissues were extracted with Trizol reagent (Catalog: 15596026; ThermoFisher Scientific) as per the manufacturer’s recommended protocol. Total RNA (1–5 μg) was treated with RNase-free DNase I (Catalog: M0303, New England Biolabs Inc.) at 37 °C for 15 min, subsequently heat-inactivated at 75 °C for 10 min and used for cDNA synthesis. The first-strand cDNA was synthesized using the SuperScript™ first-strand synthesis system (Catalog: 11904018; ThermoFisher Scientific). RT-PCR was performed in a 20 μL reaction mix containing 1× DreamTaq buffer, 2 mM dNTP mix, 0.2 μM oligonucleotide primers, cDNAs equivalent to 100 ng total RNA, and 1.25 units DreamTaq™ Hot Start DNA Polymerase (Catalog: EP1701; ThermoFisher Scientific) and amplified using 95 °C for 1 min, 35 cycles of 95 °C for 10 s, 62°-65 °C for 10 s and 72 °C for 30 s. The RT-PCR products were separated using agarose gel electrophoresis and visualized. The electrophoretically separated PCR products were gel-purified using QIAquick Gel Extraction Kit (Catalog: 28704; QIAGEN). The purified PCR products were sequenced using a dual ABI 3730XL instrument at the DNA Sequencing Facility, The Centre for Applied Genomics, The Hospital for Sick Children, Toronto, Canada.

### Blue-native polyacrylamide gel electrophoresis (BN-PAGE)

Native-PAGE under non-denaturing/reducing condition is ideal for studying multiprotein complexes [40]. In BN-PAGE, the proteins and MPCs are separated under native conditions in a first dimension (Fig. S1I). Subsequently, proteins and/or MPCs are denatured and reduced (by SDS and DTT, respectively) in the first-dimension gel strip and subjected to electrophoretic separation in the second-dimension. Component monomeric proteins will appear on a vertical line in the second-dimension corresponding to the MPCs separated in the first-dimension. The BN-PAGE was performed as described previously [40]. Briefly, the lysates were prepared by sonicating the proteins in 1x BN-PAGE lysis buffer (pH 7) containing 20 mM Bis-Tris, 500 mM 6-aminocaproic acid (Catalog: A2504, Sigma), 20 mM NaCl, 2 mM EDTA, 10% glycerol, 1.5% *n*-Dodecyl β-D-maltoside (Catalog: D4641, Sigma) and supplemented with 1X Halt protease and phosphatase inhibitor cocktail (Catalog: 1861281, ThermoFisher Scientific). The proteins were then separated in 4–15% or 10–20% gradient BN-PAGE gel using a cathode buffer (pH 7) containing 15 mM Bis-Tris, 50 mM Tricine and 0.002% Coomassie blue G250, and an anode buffer (pH 7) containing 50 mM Bis-Tris. The gel strips (individual lanes) were carefully excised including the 3.2% stacking gel and immersed in freshly prepared sample buffer containing 12.5 mM Tris (pH 6.8), 4% SDS, 20% glycerol, 100 mM DTT and 0.02% bromophenol blue, for 30 mins at 50 °C and then the proteins in the gel slices were separated in the second-dimension SDS-PAGE, immunoblotted and visualized.

### Isoelectric focusing

Fifty micrograms of total cell lysate was precipitated by acetone and dissolved in a rehydration buffer containing 8 M Urea, 2% CHAPS, 50 mM dithiothreitol (DTT) and 0.2% Bio-Lyte ampholytes pH 3–10 (Catalog: 1632094; Bio-Rad). The dissolved proteins were then incubated in a Zoom IPG 3–10 nonlinear (NL) or linear (L) strips (Catalog: ZM0011/ZM0018; ThermoFisher Scientific) for 1 h and focused at 175 V (V) for 15 min, 175-2000 V ramp for 45 min and 2000 V for 30 min respectively. After focusing, the proteins in the strips were reduced (by DTT), alkylated (by iodoacetamide) and resolved on second-dimension SDS-PAGE, immunoblotted and visualized.

### Western blotting and immune-detection and quantification

Relative quantification of proteins was done by SDS-PAGE separation of total proteins followed by transfer to a nitrocellulose membrane and immunoblotting based detection using HRP-conjugated secondary antibodies. In all Western blots equal (20-30 μg) amounts of protein were loaded for all samples in each experimental setup. In addition, same lysates were used to run two SDS-PAGE-based gels in parallel, one of them was used for immunoblotting and the other was used for Oriole staining and subsequent imaging using ChemiDoc MP Imaging System (Bio-Rad). Oriole™ fluorescent gel stain, developed by BioRad, is a highly sensitive stain for visualization and quantitation of proteins separated by SDS-PAGE [41]. The CAMKK2-CAMK4-CREB signaling [21, 27] is an important regulator of global gene expression including immediate early genes [42]. Therefore, in a study where CAMKK2 and/or CAMK4-deficient condition has been created, it would be unwise to use GAPDH or other housekeeping gene as reference gene without proper validation to quantify target proteins that are expected to be altered by CAMKK2 and/or CAMK4 deficiency. To overcome this issue, specific oriole-stained band-areas which are non-overlapping to the target protein and exhibited no visual difference in the ImageJ-based intensity plot profile were chosen and used as a reference for the relative quantification of target proteins. This approach to quantify immunoblots was extensively used in our previous studies [9, 40, 43, 44]. Also, Bio-Rad has developed a proprietary polyacrylamide gel chemistry-based system known as Stain-Free Imaging technology which is based on the similar principle of relative quantification of proteins that we used in our studies. These approaches are helpful to overcome the inherently problematic use of housekeeping proteins as loading controls on western blots, permitting the user to obtain truly quantitative western blot data by normalizing bands to total or a fraction of proteins. Table 1 summarizes all primary antibodies used in this study. The cell lysates were prepared in 1X RIPA lysis and extraction buffer (Catalog: 89900, ThermoFisher Scientific) supplemented with 1X Halt protease and phosphatase inhibitor cocktail (Catalog: 78441, ThermoFisher Scientific).

**Table 1:** Reagents and antibodies

Name	Description	Source	Catalogue number
Total Iron assay kit	Spectrometric measurement of total iron content	Sekure Chemistry	102–25
CAMKK2-CRISPR/Cas9 plasmid	gRNA and tracrRNA constructs	SCBT	SC-400928
CAMKK2-HDR plasmid	5′ and 3′ HDR sequence flanking a floxed puromycin/RFP reporter cassettes	SCBT	SC-400928-HDR
CAMK4-CRISPR/Cas9 plasmid	gRNA and tracrRNA constructs	SCBT	SC-400806
CAMK4-HDR plasmid	5′ and 3′ HDR sequence flanking a floxed puromycin/RFP reporter cassettes	SCBT	SC-400806-HDR
Anti-TF (for human)	Mouse monoclonal (Clone-E8)	SCBT	SC-393595
Anti-TF (for mouse)	Mouse monoclonal (Clone-F8)	SCBT	SC-373785
Anti-TFRC (CD71)	Mouse monoclonal (Clone-2B6)	SCBT	SC-51829
Anti-CAMKK2	Mouse monoclonal (Clone-ZZ9)	SCBT	SC-100364
Anti-CAMK4	Mouse monoclonal (Clone—A3)	SCBT	SC-166256
Anti-GAPDH	Mouse monoclonal (Clone-0411)	SCBT	SC-25778
Anti ERK1/2	Rabbit polyclonal	CST	9102
DNA sequencing primers			
Primer name	Sequence (5′-3′)		
Camkk2 Forward	TAA AGA CCA TGA TTC GAA AG		
Camkk2 Reverse	CTT TCA CAA GAG CAC TTC		

### Iron assay

The snap frozen tissue iron content was measured by the total iron assay kit (Table-1). The assay is based on ferrozine (Fz) [45] which complexed with ferrous (Fe^2+^) iron to form a tris ferrozine /iron, Fe (Fz)_3_, complex. The test is based on the principle that in an acidic medium Tf bound iron dissociates into free ferrous (Fe^2+^) and ferric (Fe^3+^) irons. Ascorbic acid is used to reduce Fe^3+^ to the Fe^2+^ state. The Fz reacts with Fe^2+^ to form a magenta Fe (Fz)_3_ complex which absorbs at 560 nm. The absorbance is directly proportional to the amount of iron. The iron content per 500 μg of total tissue protein was measured as per manufacturer’s instruction and the relative iron content was expressed in terms of absorbance at 560 nm.

### Intracellular calcium release-response assay by live-cell confocal imaging

The HEK293 cells were used to study the muscarinic signaling-mediated intracellular calcium release response because HEK293 cells endogenously express functional muscarinic acetylcholine receptors (**mAChRs**) [46, 47]. The G_Δsix0_ HEK293 cells were used to characterize the plasma membrane (**PM**) and endoplasmic reticulum (**ER**)-mediated [Ca^2+^]_i_ release response following treatment with 10 μM muscarine. In the absence of functional G-proteins, it is expected that the intracellular [Ca^2+^]_i_ release response will be affected upon agonist binding to mAChRs. The 2-aminoethoxydiphenylborane (2-APB) is a membrane-permeable inositol 1,4,5-trisphosphate receptor type 3 (**IP**_**3**_**R**) antagonist [48] which stimulates store-operated calcium (SOC) release at low concentrations (< 10 μM) and inhibits it at higher concentrations (up to 50 μM) [49]. Therefore, 50 μM 2-APB was used to inhibit ER-mediated [Ca^2+^]_i_ release. The cells were cultured for 48 h in serum-free media. The culture media was then replaced with a salt-glucose solution containing 114 mM NaCl, 0.22% NaHCO_3_, 5.29 mM KCl, with or without CaCl_2_.2H_2_O (with calcium: 2 mM CaCl_2_ + 1 mM BaCl_2_; without calcium: 3 mM BaCl_2_), 10 mM HEPES, 10 mM Glucose, 1 mM MgCl2 and supplemented with 5 μM Fluo-4 AM dye (Catalog: F14210; ThermoFisher Scientific). The cells were loaded with cell-permeant Fluo-4 for 15 min, washed and then time-series images were captured at 30 s interval following treatment with 10 μM muscarine (Catalog: M6532; Sigma). Zeiss LSM510 confocal microscope with a controlled humidified atmosphere containing 5% CO2 at 37 °C was used to capture time-lapse images. The mean Fluo-4 intensity was calculated using the ImageJ time-series analyzer plugin.

### Statistical analysis

Statistical analysis was performed using Prism version 7.00 (GraphPad Software). The mean of more than 2 groups were compared using one-way ANOVA (randomized) followed by multiple comparison tests [50, 51]. The mean of multiple experimental groups was compared with the control group by Dunnett’s post hoc multiple comparison test. Comparisons between two groups were performed using Student’s t-test (unpaired). Differences were considered significant with *P* < 0.05.

## Results

### Loss of Camk4 significantly altered Tf, Tfrc, and iron content in mouse liver and cerebellum tissues

**Fig. 1:**
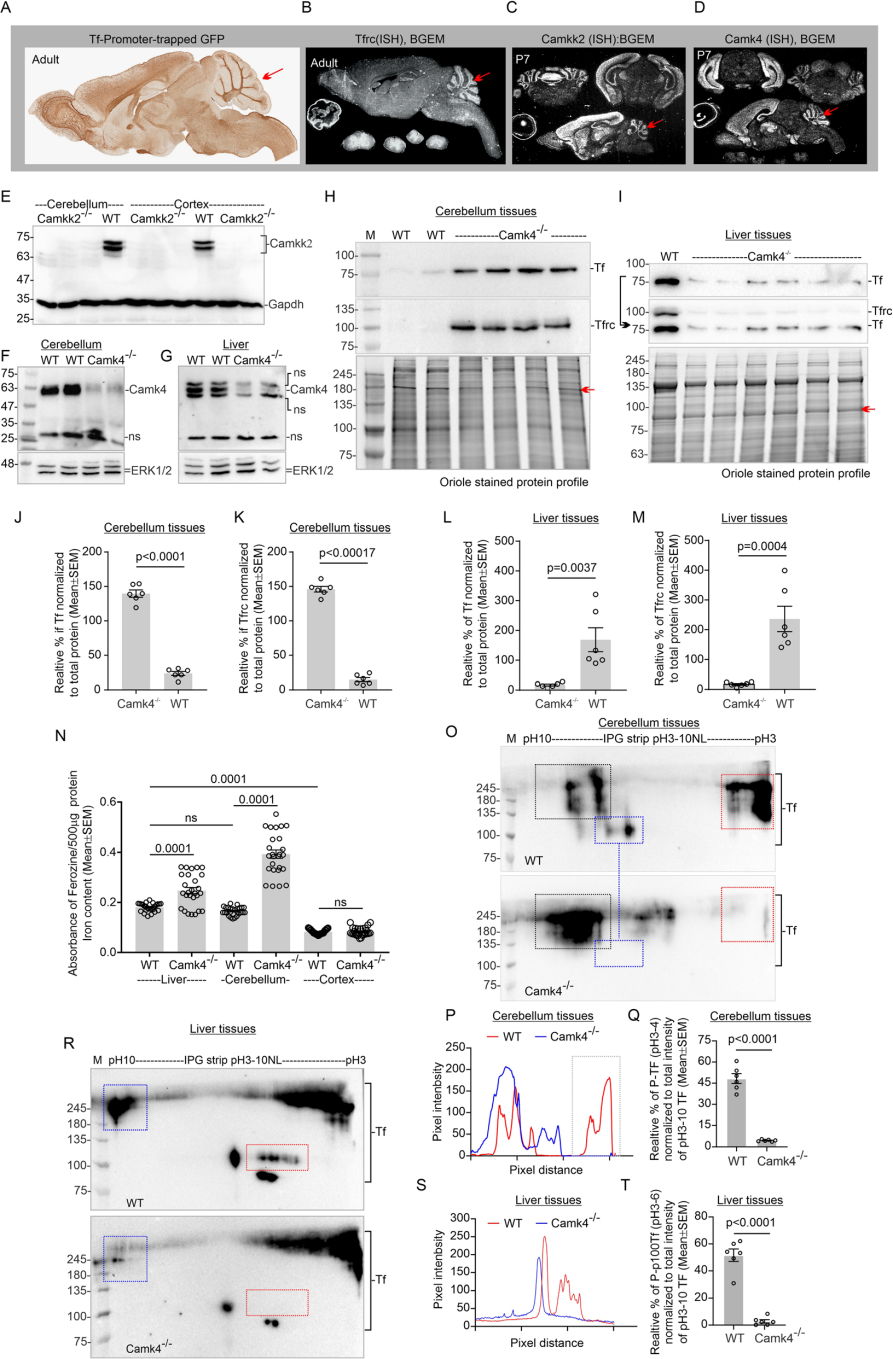
Effect of loss of Camk4 on the turnover and PTMs of Tf as well as iron content in different mouse tissues. **a**: Immunohistochemical staining of Tf promoter-trapped GFP expression in mouse brain. Data obtained from GENSAT database. B-D: RNA in situ hybridization images showing an abundance of Tfrc (**b**), Camkk2 (**c**), and Camk4 (**d**) mRNAs in mouse brain. Images obtained from BGEM database [37]. Red arrows indicate overlapping gene expressions in the cerebellum region. **e**: Immunoblots showing expression of Camkk2 and Gapdh in the cerebellum and cortex tissues form adult wild-type and Camkk2^−/−^ mice. **f**-**g**: Immunoblots showing expression of Camk4 and Erk1/2 in the cerebellum and liver tissues from adult wild-type and Camk4^−/−^ mice. Ns: nonspecific. **h**-**i**: Immunoblots showing expression of Tf and Tfrc in the cerebellum and liver tissues from wild-type and Camk4^−/−^ mice. Bottom panels represent oriole-stained gel showing total protein loading. Relative intensities of the red arrow-marked oriole-stained bands in the total protein profile were used for the quantification of Tf and Tfrc. **j**-**m**: Scatter plots showing the relative abundance of Tf and Tfrc in the cerebellum and liver tissues. *N* = 2 replicates from three wild-type and Camk4^−/−^ mice. *P* values by t-test (unpaired). **n**: Scatter plot showing iron content in different mouse tissues. *N* = 26/27, ~ 9 replicates from three wild-type and Camk4^−/−^ mice respectively. *P* values by one-way ANOVA followed by Dunnett’s multiple comparison test. **o** & **r**: Immunoblots showing charged fractions of Tf, separated by IEF/SDS-PAGE. Colored dotted rectangles indicate alterations in the differentially charged fractions of Tf. (P&S): Overlapped plot profiles of the immunoblots presented in **o** & **r** respetively. In the cerebellum, p100-245 kDa Tf spots were considered for plot profiling. In the liver tissue, p75-100 kDa Tf spots were considered for plot profiling. **q** &**t**: Scatter plots showing the relative amount of negatively charged fraction of Tf (red rectangle areas). *N* = 6, 2 replicates from three wild-type and Camk4^−/−^ mice respectively. *P* values by t-test (unpaired).

Tf, Tfrc, Camkk2, and Camk4 have an overlapping expression in mouse brain, specifically in the cerebellum region as revealed by the promoter-trapped reporter (green fluorescent protein: **GFP**) expression documented in GENSAT-based [36] study and ISH-based studies in BGEM project [37] (Fig. 1a-d, red arrows). Immunoblotting revealed that Camkk2 and Camk4 are expressed in mouse brain, specifically in the cerebellum (Fig. 1e-f). Camkk2 proteins appeared as two major bands in the range of p70–75 kilodaltons (**kDa**) in both cortex and cerebellum which are absent in the Camkk2^−/−^ mice (Fig. 1e). The p70–75 Camkk2 proteins may be due to alternative splicing involving exon 16. Alternative splicing of the CAMKK2 exon 14 and/or 16 in human glioblastoma/astrocytoma cells [52] and exon 16 in rat neuroblastoma cells [53] has been reported. The theoretical molecular mass of Camkk2^+ 16^ and Camkk2^Δ16^ (Δ: skipped) isoforms (GenBank ID: NM_001199676.1 and NM_145358.2 respectively) are 64.6 and 59.6 kDa respectively (Fig. S2A-B). In addition to the existence of transcriptional variants, it is also possible that p70–75 kDa Camkk2 proteins may in part due to the PTM that added considerable molecular mass, for example, ubiquitination of CAMKK2 at multiple residues have been reported by high throughput mass spectrometric analysis as archived in the PhosphoSitePlus database [54, 55].

To verify alternative splicing of Camkk2 exon 16 in mouse tissues, two sets of polymerase chain reaction (PCR) primers were designed to amplify 270/242 and 227/199 base-pair (bp) amplicons corresponding to Camkk2^+ 16^ and Camkk2^Δ16^ isoforms respectively (Fig S2AB). The PCR amplicon sizes were calculated based on the reference sequences (NM_001199676.1 and NM_145358.2) available in the GenBank database. RT-PCR using total RNA extracted from the wild-type mouse cortex, cerebellum and liver tissues revealed a tissue-specific differential expression pattern of Camkk2 isoforms as revealed by PCR amplicons equivalent to ~ 200, ~ 250 and ~ 300 bp products (Fig S2CD). To understand the nature of the PCR amplicons, amplified products from the liver tissue were gel-purified (Fig S2EF) and sequenced (Fig S2GH) using two nested sequencing primers (Table 1). BLAST-like alignment tool (BLAT)-based pairwise alignment of the DNA sequence derived from the ~ 300 bp PCR product (Fig. S3A) revealed insertion of a 41 bp sequence after exon 14 (Fig. S3B&E-F), partial skipping of exon 15 (Fig. S3C&E-F), insertion of an 18 bp sequence after exon 15 (Fig. S3E-F), complete skipping of exon 16 (Fig. S3A) and a gain of 3 nucleotides in the splice acceptor site of exon 17 (Fig. S3D&E-F) of Camkk2 gene. Thus, the ~ 300 bp product turned out to be an exon 16 skipped (Camkk2^Δ16^) isoform which gained an additional 69 bp sequence thus shifting the band to an area corresponding to a higher DNA size which was not anticipated. This unique splicing pattern would prematurely terminate CAMKK2^Δ16^ reading frame at 516 amino acid residue by generating a stop codon (Fig. S3F). As the gel-purified products were sequenced directly without sub-cloning, there was a possibility of heterogeneous amplicons which is reflected in the presence of minor peaks in the chromatogram (Fig. S3G). Based on the chromatogram, a “TTC” allele instead of a “TAG” allele would replace the stop codon at 516 residue with phenylalanine and generate a downstream alternative stop codon (TAA) at 527 amino acid residue in CAMKK2^Δ16^ isoform (Fig. S3H). BLAT alignment of the DNA sequence derived from the ~ 200 bp product revealed an identical sequence alignment with CAMKK2^+ 16^ (NM_001199676) isoform (Fig. S1H and S4). The diffused nature of the intermediate ~ 250 bp bands (Fig. S2F) generated non-specific sequences in the chromatogram and may represent PCR artifacts (data not shown). Based on these data, it can be concluded that the expression of CAMKK2^Δ16/+ 16^ isoforms corresponds to multiple protein bands observed in the range of p70–75 kDa. These results warrant a detailed analysis of the Camkk2 isoforms in tissue and cell-type-specific manner in the future to understand the variations in Camkk2 protein products. Recently, we characterized alternative splicing of CAMKK2 exon 14 in EA.hy926 human endothelial cells (manuscript in revision in BBA-Molecular Cell Research) which correlated with the appearance of p70–75 CAMKK2 proteins in multiple human cell lines including HEK293 and HepG2 cells [9]. Thus, it is justified to consider p70–75 kDa protein bands in mouse and human transformed cells as CAMKK2 transcriptional isoforms.

Camk4 appeared as a ~ 63 kDa protein in the wild-type cerebellum and liver tissues which is absent in corresponding tissues in Camk4^−/−^ mice (Fig. 1f-g). Immunoblotting-based quantification revealed a significantly high amount of Tf and Tfrc present in Camk4^−/−^ cerebellum tissues compared to the wild-type (Fig. 1h, j-k). The cerebral cortex tissue exhibited no significant difference in the abundance of Tf (p75) and Tfrc (Fig. S5A-D). In contrast to the cerebellum, a significant reduction of Tf and Tfrc was observed in Camk4^−/−^ liver tissues compared to the wild-type (Fig. 1i, l-m). Ferrozine-based iron quantification assay revealed a significantly increased iron content in the cerebellum and liver tissues in Camk4^−/−^ mice compared to the wild-type (Fig. 1n). The significantly increased iron content and increased level of Tf and Tfrc in Camk4^−/−^ cerebellum positively correlated, whereas, increased iron content negatively correlated with decreased Tf and Tfrc levels in Camk4^−/−^ liver tissues.

In a previous study, [9] based on isoelectric focusing (**IEF**), it was reported that loss of Camkk2 leads to a significant reduction in the pH ~ 3–4 fraction of Tf in different parts of mouse brain and liver tissues as well as in cultured primary rat dorsal root ganglion (DRG) neurons, HEK293 and HepG2 cells [9]. To study the effect of Camk4 loss-of-function on the PTMs (charged fractions) of Tf, total proteins were subjected to IEF followed by second-dimension SDS-PAGE and immunoblotting (Fig. 1o&r). The theoretical molecular weight of mouse Tf (UniProt ID: Q921I1) is **76 kDa** and isoelectric point (pI) is **6.9**, calculated by ExPASy Compute pI/Mw tool [56]. In the cerebellum, Tf appeared as multiple high molecular weight (> 100 kDa) charged fractions which exhibited a considerable difference between the wild-type and Camk4^−/−^ mice (Fig. 1o, colored rectangles). A plot profile-based quantification (Fig. 1p) revealed a significantly reduced pH ~ 3–4 fraction of Tf in the cerebellum of Camk4^−/−^ mouse compared to the wild-type (Fig. 1q). In the liver, Tf also appeared as multiple charged fractions at p75kDa and higher molecular weight fractions, which exhibited a considerable difference between Camk4^−/−^ and wild-type mice (Fig. 1r). A plot profile-based quantification (Fig. 1s) of the p100 Tf revealed a significantly decreased pH ~ 3–6 fraction of Tf in Camk4^−/−^ liver tissue compared to the wild-type (Fig. 1t).

### CRISPR/Cas9-mediated genetic ablation of CAMKK2, CAMK4, and CAMKK2 + CAMK4 significantly increased receptor-mediated TF uptake in HEK293 cells

**Fig. 2:**
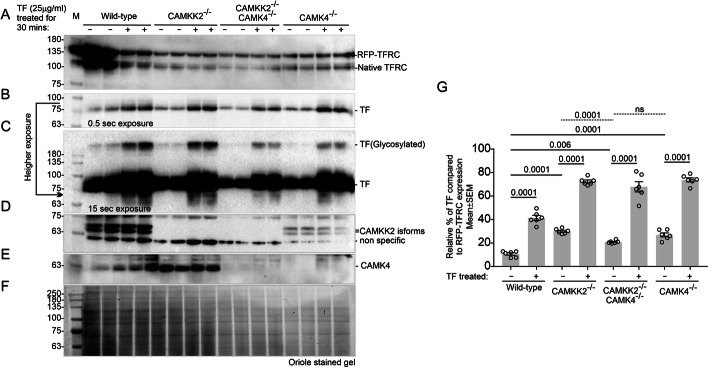
Loss of CAMKK2 and/or CAMK4 significantly increased TF-uptake by the HEK293 cells. **a**-**e**: Immunoblot showing relative expression/abundance of RFP-TFRC (**a**), TF(**b**-**c**), CAMKK2 (**d**), CAMK4(**e**) in the wild-type, CAMKK2^−/−^, CAMK4^−/−^, and DKO HEK293 cells treated with 25 μg/ml partially iron-saturated human TF. **f**: Oriole-stained gel showing total protein loading. **g**: Scatter plot showing the relative amount of TF. N = 6, 2 replicates from three independent experiments. *P* values by one-way ANOVA test followed by multiple comparisons.

The role of CAMKK2-CAMK4 signaling in receptor-mediated TF trafficking was studied by using CAMKK2^−/^^−^[9], CAMK4^−/−^ and CAMKK2^−/−^/CAMK4^−/−^ (**DKO**) HEK293 cell clones, generated by CRISPR/Cas9-based targeted gene deletion of respective genes (Fig. S1A-H). Immunoblotting-based quantification revealed a significantly increased TF uptake within 1 h of treatment by serum-starved CAMKK2^−/−^, CAMK4^−/−^ and DKO HEK293 cell clones compared to the wild-type, all transiently transfected with a red fluorescent protein (**RFP**)-tagged TFRC (Fig. 2a-g). Also, the basal TF level was found significantly high in the CAMKK2^−/−^, CAMK4^−/−^, and DKO HEK293 cell clones compared to the wild-type (Fig. 2g).

### CAMK4^−/−^ and DKO HEK293 cells retained abnormally high membrane-associated TF following pulse-treatment

**Fig. 3:**
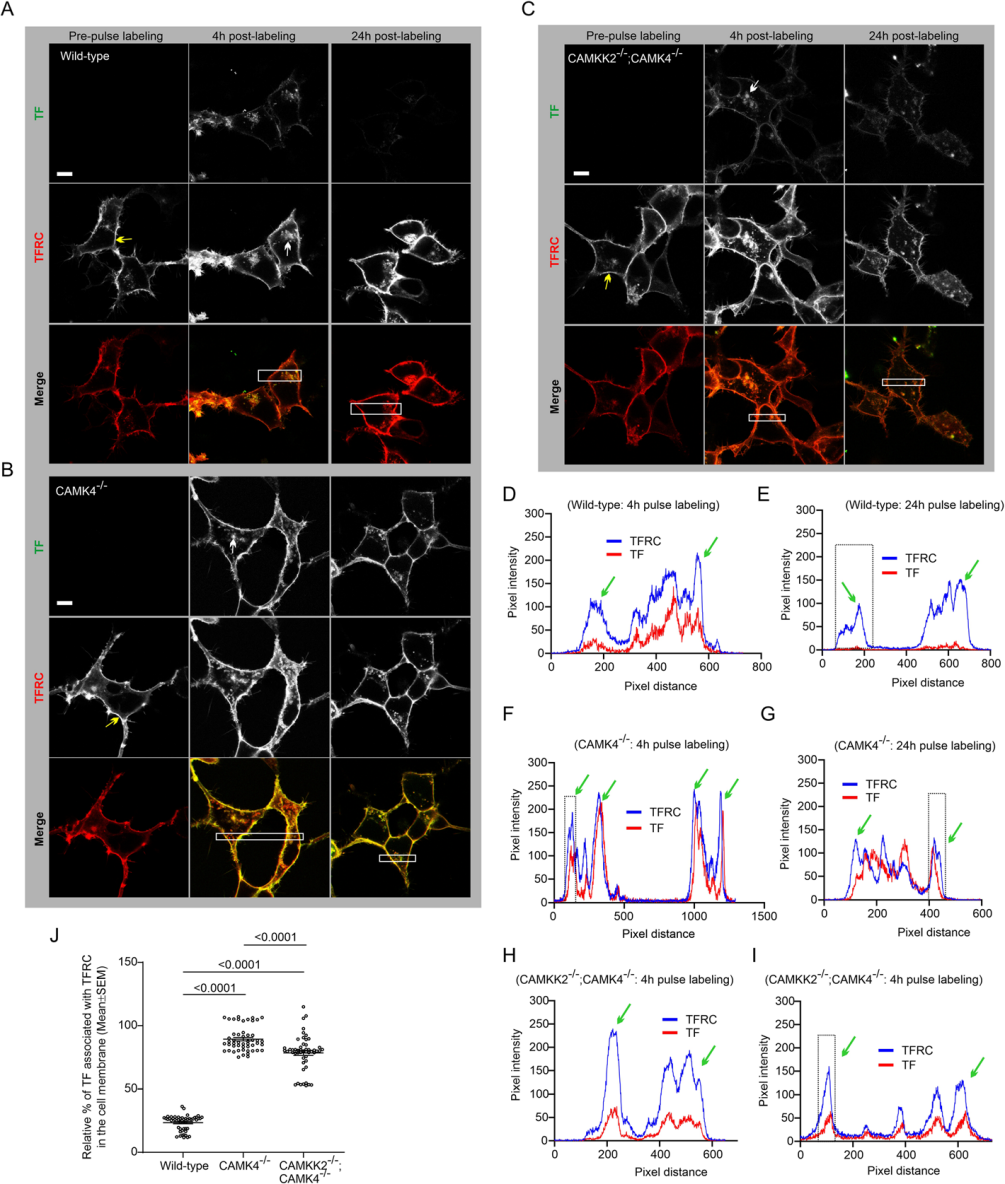
Aberrant TF trafficking by CAMK4^−/−^ and DKO HEK293 cells. **a-c**: Live confocal fluorescent images showing localization of FITC-TF and RFP-TFRC in wild-type, CAMK4^−/−^ and DKO HEK293 cells. The cells were pulse-labeled with 25 μg FITC-TF for 1 h followed by a triple wash in 1xPBS buffer and then incubated in serum-free media. Following pulse-labeling, cells were imaged at 4 h and 24 h. The yellow arrows indicate membrane localization of RFP-TFRC and white arrows indicate internalized TF in vesicular structures. The white rectangle area indicates the region used for plot profiling using ImageJ software. Scale: 10 μm. **d**-**i**: Plot profiles of TF and RFP-TFRC intensities in the white rectangular areas marked in figure A-C respectively. Green arrows indicate the peaks representing membrane-localized RFP-TFRC/FITC-TF. The black dotted rectangular areas in Figs. E, G and J represent the peak areas used in determining the relative amount of TFRC/TF associated with the membranes. **j**: Scatter plot showing the relative amount of FITC-TF associated with membrane-bound RFP-TFRC after 24 h of pulse-labeling. *N* = 51, ~ 10 replicates from 5 independent experiments. *P* values by one-way ANOVA test followed by multiple comparisons.

FITC-conjugated TF uptake was studied by pulse-treatment of RFP-TFRC transfected wild-type, CAMK4^−/−^, and DKO HEK293 cells with of 25 μg/ml FITC-TF for one hour. Live-cell confocal imaging revealed the membrane localization of TFRC (Fig. 3a-c, yellow arrows). Plot profile-based relative quantification (Fig. 3a-c & d-i, green arrows) revealed a significantly increased amount of FITC-TF associated with the membrane-localized RFP-TFRC in CAMK4^−/−^ and DKO cells compared to the wild type following 24 h of pulse-treatment (Fig. 3j). Also, membrane retention of TF was found significantly decreased in DKO cells compared to the CAMK4^−/−^ cells (Fig. 3j).

### CAMKK2 and CAMK4 are associated in a 146 kDa multiprotein complex (MPC) in HEK293 cells

**Fig. 4:**
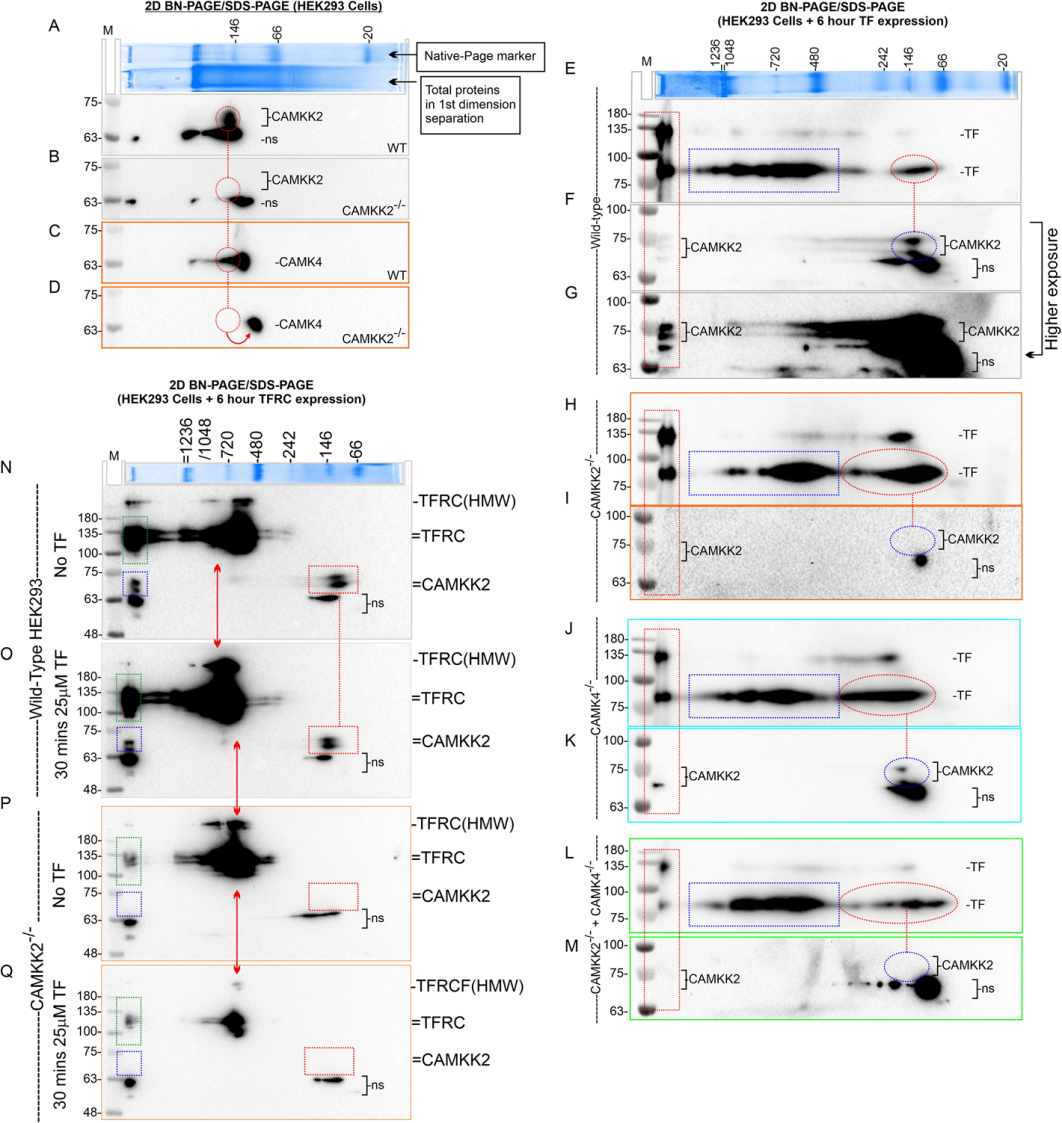
Alterations in CAMKK2, CAMK4, TF, and TFRC-associated MPCs. **a**-**d**: Immunoblots showing CAMKK2 (**a**-**b**) and CAMK4-associated MPCs (**c**-**d**) in the wild-type (WT) and CAMKK2^−/−^ HEK293 cells. The red circles represent the vertical alignment of ~ 146 kDa CAMKK2 and CAMK4-associated MPCs which indicates the possibility that these proteins occupied the same MPCs. The red arrow in D indicates that CAMK4-associated MPCs shifted to a lower molecular weight complex in the absence of CAMKK2 which is probably due to the loss of an interacting partner. The top Coomassie-stained gel slice is showing the separation of native-PAGE markers in the first-dimension along with the separation of total cellular proteins in the next lane. The MPCs presented in the A-D were separated in the same first-dimension BN-PAGE, therefore, relative migration of the MPCs are comparable in the second-dimension SDS-PAGE. The nonspecific (ns) band in the anti-CAMKK2 immunoblot is persistence in the CAMKK2-deficient cells indicating non-specific binding of the antibody. **e**-**m**: Immunoblots showing alterations in the endogenously expressed TF and native CAMKK2-associated MPCs in the wild-type, CAMKK2^−/−^, CAMK4^−/−^ and DKO HEK293 cells. The cells were transiently transfected with TF for 6 h and the protein lysates were subjected to BN-PAGE/SDS-PAGE analysis. Red rectangles (E-M) indicate the vertical alignment of CAMKK2 and TF-associated > 1200 kDa MPCs, which is dramatically reduced in DKO cells (**l**). Blue rectangles (**e**, **h**, **j**, and **l**) indicate multiple TF-associated MPCs in the range of 480–1200 kDa which exhibited considerable difference in CAMKK2-deficient cells. Red circles (**e**, **h**, **j**, and **l**) indicate a ~ 146 kDa TF associated MPC that is differentially shifted to higher molecular weight complexes in CAMKK2^−/−^, CAMK4^−/−^ and DKO cells. Blue circles (**f**, **i**, **k**, and **m**) indicate CAMKK2-associated ~ 146 kDa MPCs vertically aligned with the TF-associated MPCs. The loss of CAMKK2 and/or CAMK4 caused a considerable shift in the ~ 146 kDa TF-associated MPCs which indicates potential functional dependency between these proteins. **n**-**q**: Immunoblots showing alterations in the CAMKK2- and TFRC-associated MPCs in the serum-starved wild-type and CAMKK2^−/−^ HEK293 cells treated with or without 25 μg/ml TF for 30 mins. The immunoblots presented here were first immunoblotted using anti-TFRC antibody and subsequently immunoblotted using anti-CAMKK2 antibody to detect co-migration of TFRC and CAMKK2 associated protein complexes respectively. The anti-TFRC immunoblots were presented in Fig. 5 to exhibit co-migration of TFRC with TF-associated proteins. Native TFRC is an 84kda protein but a 100 kDa TFRC and 135 kDa RFP-TFRC is visible in the immunoblots. In addition, > 135 kDa TFRCs were also present. All high molecular fraction of TFRC may be due to PTMs because the denatured (50 °C) and reducing (100 mM DTT) second-dimension SDS-PAGE would disrupt any potential dimers or oligomers formation due to intermolecular disulfide-bridge. Green and blue rectangles indicate the vertical alignment of > 1200 kDa TFRC/RFP-TFRC and CAMKK2-associated MPCs. The relative amount of > 1200 kDa TFRC-associated MPCs was comparatively reduced in CAMKK2^−/−^ cells indicating potential functional implication and interdependency. Red arrows indicate a relative shift of > 480 kDa TFRC-associated MPCs in untreated and TF-treated wild-type and CAMKK2^−/−^ cells. Red rectangles indicate CAMKK2-associated 146 kDa MPCs. “-” means “without TF treatment”; “+” means “with TF treatment”; “=” means “two bands of the same protein”.

The protein-protein interactions involving CAMKK2, CAMK4, TF, and TFRC was studied to pinpoint the defect in TF-trafficking under loss of CAMKK2-CAMK4 signaling. There are multiple approaches to studying protein-protein interaction, each with limitations [57]. Native gel electrophoresis is an efficient technique to study multi-protein interactions where both covalent and electrostatic interactions are highly preserved [58, 59]. Previously, using two-dimensional **BN-PAGE** followed by SDS-PAGE-based technique, CAMKK2 was found associated with a > 1200 kDa and 146KDa MPCs [9]. Therefore, BN-PAGE/SDS-PAGE (Fig. S1I) was used to study alterations in the interaction of CAMKK2, CAMK4, TF, and TFRC associated protein complexes. Vertical alignment of the MPCs associated with different proteins in the BN-PAGE/SDS-PAGE indicates their potential association in a single complex (Fig. S1I). BN-PAGE revealed that both CAMK4 and CAMKK2 are associated in a ~ 146 kDa MPC in the wild-type HEK293 cells (Fig. 4a-c, Red circles). This is further supported by the observation that loss of CAMKK2 shifted CAMK4-associated ~ 146 kDa MPC to a ~ 100 kDa complex as anticipated (Fig. 4d, red circle, and arrow).

### Loss of CAMKK2 and/or CAMK4 altered endogenous TF associated MPCs in HEK293 cells

TF was transiently transfected in the wild-type, CAMKK2^−/−^, CAMK4^−/−^, and DKO HEK293 cells to study endogenous TF and CAMKK2 associated MPCs (Fig. 4e-m). TF appeared as multiple MPCs at > 1200 kDa, 480–1200 kDa and ~ 146 kDa in the wild-type cells (Fig. 4e, colored rectangles, and the circle). The 146 kDa and > 1200 kDa TF-associated MPCs were vertically aligned with the corresponding CAMKK2-associated MPCs indicating potential interaction between these proteins (Fig. 4e-g, red rectangle and colored circles connected by dotted line). Loss of CAMKK2 altered TF-associated MPCs, specifically in the 480–1200 kDa range (Fig. 4e&h, blue rectangles). The 146 kDa TF-associated MPCs shifted to ~ 146–300 kDa range in CAMKK2^−/−^ cells with a relatively increased abundance compared to the wild-type cells (Fig. 4e&h, red circles). Loss of CAMK4 also shifted ~ 146 kDa TF-associated MPCs to ~ 146–300 kDa range with an increased abundance compared to the wild-type cells (Fig. 4e&j, red circles). Interestingly, the combined loss of CAMKK2 and CAMK4 caused a dramatic reduction of > 1200 kDa TF-associated MPCs (Fig. 4e&l, red rectangle). Also, the ~ 146 kDa TF-associated MPCs were relatively reduced in the DKO cells compared to the CAMKK2^−/−^ or CAMK4^−/−^ cells (Fig. 4h, j&l, red circles). Overall, this result indicates that CAMKK2 and CAMK4 control endogenous TF-associated MPCs possibly by recruiting or de-recruiting interacting proteins. Native TF is a 77 kDa protein, but multiple high molecular weights (**HMW**) TF were observed in the second-dimension SDS-PAGE which may be due to PTMs.

### Loss of CAMKK2 altered transiently expressed TFRC-associated MPCs

BN-PAGE/SDS-PAGE analysis of the transiently expressed RFP-TFRC in wild-type HEK293 cells revealed that TFRC is present as several MPCs in the ~ 480–1200 kDa range and a discrete > 1200 kDa MPC (green rectangle) in untreated cells (Fig. 4n). Within 30 mins of TF treatment, the TFRC-associate MPCs in the wild-type cells shifted to a higher molecular weight range starting from ~ 720 kDa (Fig. 4o, red arrow). This indicates the recruitment of TF and other interacting proteins to the TFRC-associated MPCs. Also, TF treatment caused the appearance of HMW (> 135 kDa) fractions of TFRC in the second-dimension SDS-PAGE, which may be due to PTMs (Fig. 4o). In the same immunoblots, CAMKK2 vertically aligned with the discrete > 1200 kDa TFRC-associated MPCs indicating a potential association between these proteins in these MPCs (Fig. 4no, blue and green rectangles). The ~ 146 kDa CAMKK2-associated MPC was not associated with TFRC (Fig. 4no, red rectangles). Similar experiments revealed a dramatic reduction of the > 1200 kDa TFRC-associated complex in the untreated (basal) as well as TF-treated CAMKK2^−/−^ cells (Fig. 4pq, green and blue rectangles). Also, upon TF treatment, the ~ 480–720 kDa TFRC-associated MPCs remained static (Fig. 4pq, red arrows), not shifted to the higher molecular weight regions, which indicated a lack of recruitment of interacting proteins in this MPCs in CAMKK2-deficient cells. Overall, these findings indicate that CAMKK2 regulates TFRC-associated MPCs.

### Loss of CAMKK2 and/or CAMK4 altered TF and TFRC-associated MPCs during TF trafficking

**Fig. 5:**
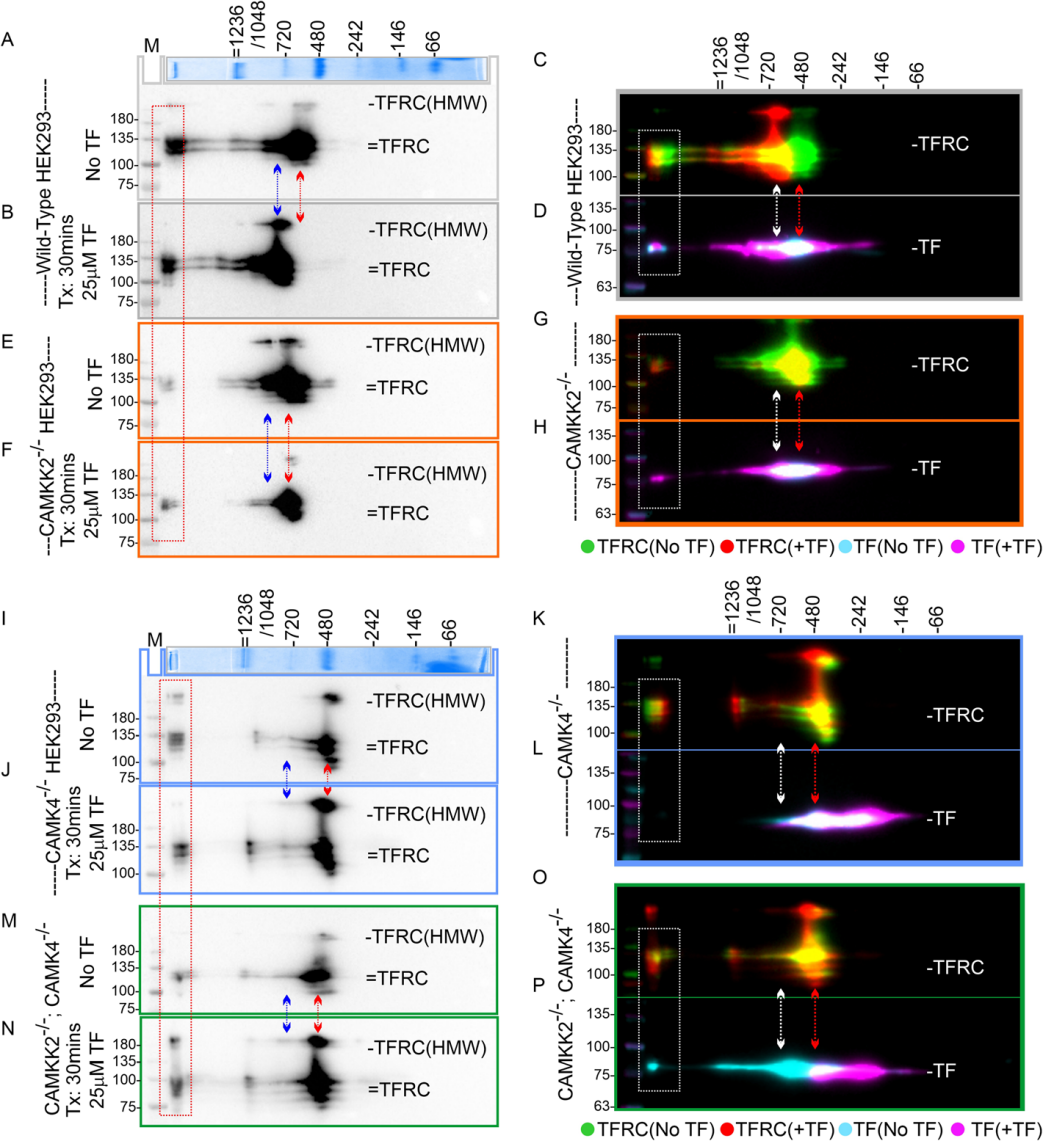
Alteration of TF and TFRC-associated MPCs during trafficking in the wild-type, CAMKK2^−/−^, CAMK4^−/−^, and DKO HEK293 cells. (**ab**+**ef** & **ij** + **mn**): Immunoblots showing alterations of TFRC-associated MPCs in serum-starved wild-type (**a**-**b**), CAMKK2^−/−^ (**e**-**f**), CAMK4^−/−^(**i**-**j**) and DKO (**m**-**n**) HEK293 cells transiently-transfected with RFP-TFRC for 6 h and treated with or without 25 μg/ml TF for 30 mins. The immunoblots representing different knockout cell clones are color-coded. The MPCs in each second-dimension-derived immunoblot were separated together in the same first-dimension BN-PAGE. The immunoblots are aligned to show relative migration of the protein complexes. Red rectangles (**ab**+**ef** & **ij** + **mn**) indicate the vertical alignment of > 1200 kDa TFRC-associated MPCs, which is dramatically reduced in CAMKK2^−/−^, CAMK4^−/−^, and DKO cells. Red arrows indicate the relative position of native ~ 480–720 kDa TFRC-associated MPCs. Blue arrows showing the relative shift in native ~ 480–720 kDa TFRC-MPCs in TF-treated CAMKK2^−/−^, CAMK4^−/−^, and DKO cells. The Coomassie-stained native page markers provided at the top of each group of immunoblots (AB&EF/ IJ&MN) indicate proteins separated in the same first dimension BN-PAGE. Tx: treatment with TF. (**cd** + **gh** & **kl** + **op**): The anti-TFRC immunoblots from **ab**+**ef** & **ij** + **mn** were false colored and overlaid with corresponding anti-TF immunoblots to show co-migration of the TF/TFRC-associated MPCs during trafficking in the TF-treated/untreated CAMKK2 and/or CAMK4-deficient cells. The MPCs within each color-coded (grey/orange/blue/green) panels were separated in the same first-dimension BN-PAGE; therefore, their relative shift in position is comparable. Vertical alignment indicates potential association between the same MPCs. White arrows indicate a relative shift in the TF and TFRC-associated MPCs. The anti-TFRC immunoblots presented in Fig. 5a, b, e, and f were also presented in Fig. 4 n-q after immunoblotting with anti-CAMKK2 antibody. “No TF”: without TF treatment; “+TF”: treatment with TF. White and red arrows showing the relative shift in native ~ 480–720 kDa TFRC-MPCs in TF-treated CAMKK2^−/−^, CAMK4^−/−^, and DKO cells. White dotted rectangles indicate the vertical alignment of > 1200 kDa TF/TFRC-associated MPCs, which is dramatically reduced in CAMKK2^−/−^, CAMK4^−/−^, and DKO cells.

To study TF and TFRC-associated MPCs during TF trafficking, the transient RFP-TFRC expressed wild-type, CAMKK2^−/−,^ CAMK4^−/−^ and DKO HEK293 cells were serum-starved overnight and subjected to TF treatment for 30 mins followed by BN-PAGE/SDS-PAGE-based analysis of the MPCs. In the wild type cells, basal TF and TFRC-associated complexes perfectly aligned (vertically) in 480 kDa and > 1200 kDa MPCs as observed before (Fig. 5a, c-d, red arrows). Upon TF treatment, the 480 kDa TF- and TFRC-associated MPCs co-migrated to the higher molecular weight complexes in the range of 720–1200 kDa as expected (Fig. 5a-d, blue and white arrows). This indicated the recruitment of interacting proteins. In CAMKK2^−/−^ and CAMK4^−/−^ cells, the basal TF/TFRC-associated MPCs also vertically aligned (red arrows) in a ~ 480 kDa MPC but remained static upon TF treatment (Fig. 5e-h & i-l, white and blue arrows). Interestingly, in CAMK4^−/−^ cells, a fraction of TF associated MPCs shifted to a lower molecular weight (< 242 kDa) regions which were not observed in CAMKK2^−/−^ cells (Fig. 5h&l), this indicates that these two kinases have a specific role in TF trafficking which may differ in terms of recruitment of interacting proteins. Also, in TF treated DKO cells, the ~ 480 kDa TF/TFRC-associated MPCs (red arrows) remained static (Fig. 5 m-p, colored arrows) and only the TF-associated MPCs showed some movement by shifting to a lower molecular weight (< 242 kDa) regions compared to the CAMKK2^−/−^ cells (Fig. 5h&p). This again reinforces the previous conclusion that these two kinases have a specific effect on TF/TFRC-associated MPCs by recruiting or dissociating possibly different sets of interacting proteins. Overall, these results indicate a complex interplay of CAMKK2 and CAMK4 in regulating TF and TFRC-associated MPCs during trafficking.

The TFRC overexpression-based experiments provided evidence for the efficacy of 2D BN-PAGE/SDS-PAGE in studying the recruitment of potential interacting proteins (TF) to the TFRC-associated MPCs. It was demonstrated that TF and TFRC-associated protein complexes co-migrated to higher molecular weight regions in TF treated cells and this shift disappeared in CAMKK2 and/or CAMK4-deficient cells suggesting failure to recruit interacting proteins. The reason for using an overexpression-based system was that HEK293 cells express a low amount of TFRC when grown in DMEM+ 10%FBS (Fig. S6A), and therefore, not very effective to perform 2D-BN-PAGE/SDS-PAGE-based analysis which require a higher amount of target protein loading. Therefore, to demonstrate the co-migration of TF and TFRC-associated MPCs under constitutive native gene expression condition, HEK293 cells were grown in Opti-MEM I reduced serum media (ThermoFisher) supplemented with 5% FBS for 72 h. The Opti-MEM media contains human Cohn fraction IV [60] paste-derived TF and other uncharacterized serum protein factors. Mass spectrometric analysis of Opti-MEM media revealed presence of a variety of growth factor proteins which may be co-precipitated along with TF during Cohn fractionation (data not shown). Interestingly, Opti-MEM was found effective to increase native TFRC expression within 24–72 h of culture compared to DMEM+ 10%FBS growth condition (Fig. S6A). BN-PAGE/SDS-PAGE was performed using 72 h Opti-MEM-grown cells treated with or without 25 μg/ml TF for 30 mins (Fig. S6B-G). Constitutively expressed native TFRC appeared as two major protein complexes at ~ 480 kDa (red rectangle) and > 1200 kDa (Fig. S6B). TF treatment shifted ~ 480 kDa TFRC-associated complex to a comparatively high molecular weight region as anticipated (Fig. S6C-D, blue rectangle). In addition to native 100 kDa TFRC, a HMW (~ 120 kDa) fraction of TFRC observed in the second-dimension SDS-PAGE which agreed with the similar observation in overexpression-based study (Fig. 5). To demonstrate the co-migration of TF with the TFRC-associated complexes, the immunoblots were treated with an anti-TF antibody without stripping and subsequently visualized (Fig. S6E-F). TF treatment caused the co-migration of both TF and TFRC-associated ~ 480 kDa complexes (red rectangle) to a comparatively HMW region (blue rectangle) suggesting recruitment and co-migration of TF and other interacting proteins to the TFRC-associated complex in TF-treated cells (Fig. S6F-G).

### Loss of CAMKK2 and CAMK4 differentially altered the charged fractions (PTMs) of TFRC in HEK293 cells

**Fig. 6:**
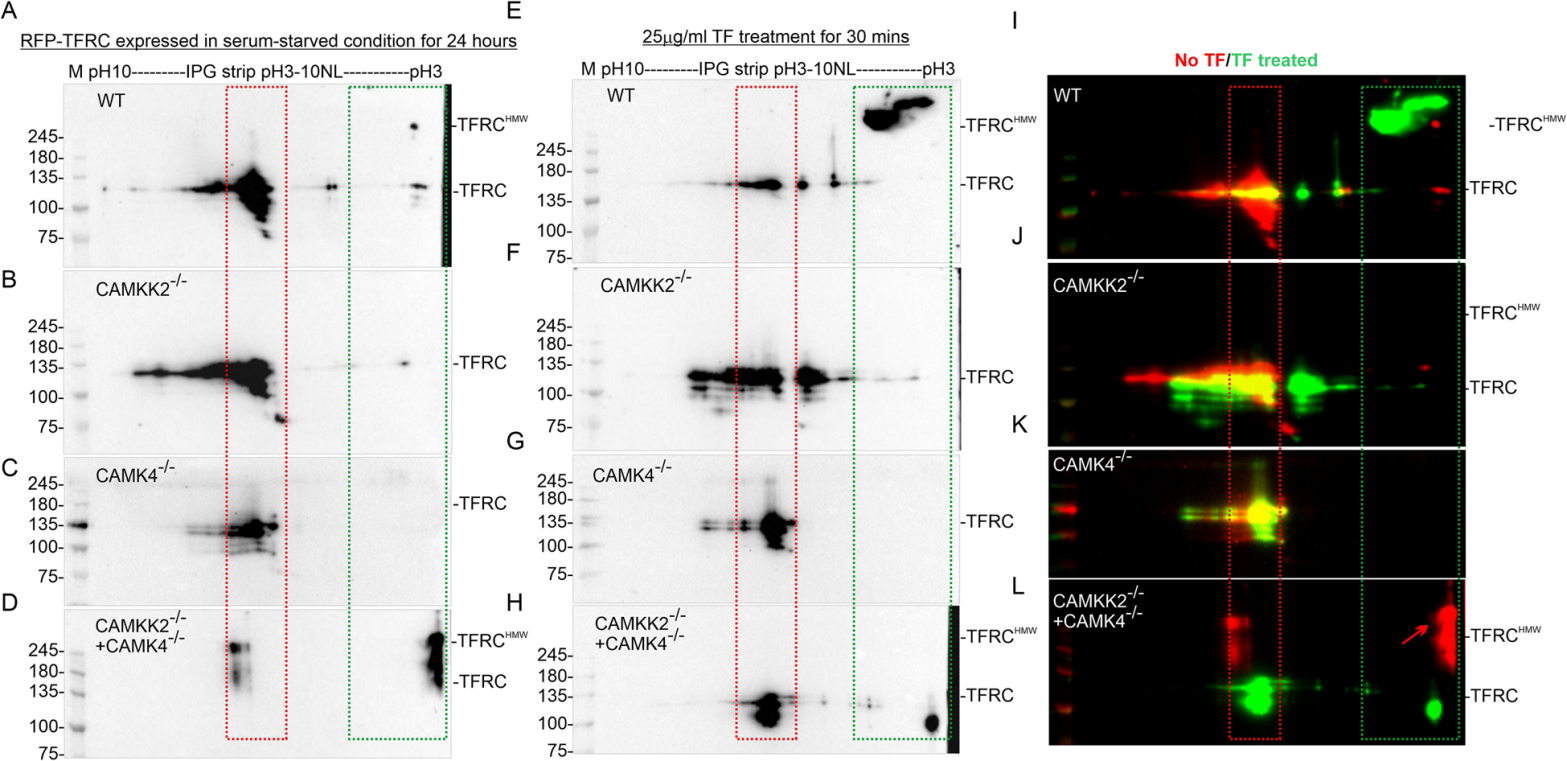
Effect of CAMKK2 and CAMK4 loss on the charged fractions (PTMs) of TFRC during TF trafficking. **a**-**h**: Immunoblots showing IEF/SDS-PAGE-based charge-separated TFRC in serum-starved wild-type (AE), CAMKK2^−/−^ (BF), CAMK4^−/−^(CG) and DKO (DH) HEK293 cells. Cells were transiently transfected with RFP-TFRC for 6 h and then treated with or without 25 μg/ml TF for 30 mins. I-L: The immunoblots in A-D and E-H were false-colored and overlaid to show the relative shift of TFRC charged fractions following TF treatment. The red rectangle indicates a major fraction of the TFRC at pH 6–7 (TFRC pI: 6.18). The green rectangle indicates acidic fractions (pH ~ 3–4). HMW: High molecular weight fraction.

Previous study showed that CAMKK2 affected TF PTMs [9], however, PTMs of TFRC were not studied. The altered TF and TFRC-associated MPCs may be due to differential PTMs of TFRC. Multiple phosphorylation, acetylation, and ubiquitination of TFRC have been documented in the archives of the PhosphositePlus database [54, 55]. Therefore, alterations in the PTMs (charged fractions) of TFRC were studied by IEF/SDS-PAGE. Total proteins from the RFP-TFRC expressed cells, treated/untreated with TF, were subjected to IEF/SDS-PAGE followed by immunoblotting using the anti-TFRC antibody. The isoelectric point of TFRC is 6.18 (Uniport ID: P02786). IEF/SDS-PAGE revealed that basal (not treated with TF) TFRC exist as a major charged fraction at pH/pI~ 6–7 (red square) and some minor fractions at pH ~ 3–6 (green square) in the wild-type serum-starved cells (Fig. 6a). However, upon TF treatment majority of the TFRC in the wild-type cells shifted to a > 245 kDa pH ~ 3–4 fraction which indicates PTMs that contributed both charge and mass (Fig. 6e&i, green square). Interestingly, loss of CAMKK2, CAMK4, and CAMKK2 + CAMK4 differentially affected the charged fractions of TFRC in untreated as well TF-treated cells (Fig. 6b-d, f-h &j-k, colored rectangles). The high molecular weight fractions of TFRC may arise due to PTMs that add additional mass to the protein, for example, mono-ubiquitination adds a molecular mass of 8.5 kDa [61] whereas phosphorylations can shift the pI by several pH units [62]. Multiple phosphorylated and ubiquitinated residues in TFRC have been documented in the archives of the PhosphositePlus database [54, 55]. Overall, these results indicate that CAMKK2 and CAMK4 control a wide variety of TFRC PTMs which in turn may control their association/disassociation with interacting proteins during receptor-mediated TF trafficking which is reflected in the BN-PAGE-based study.

### CAMKK2-CAMK4 signaling cascade regulates ER-mediated Ca^2+^ release in HEK293 cells

**Fig. 7:**
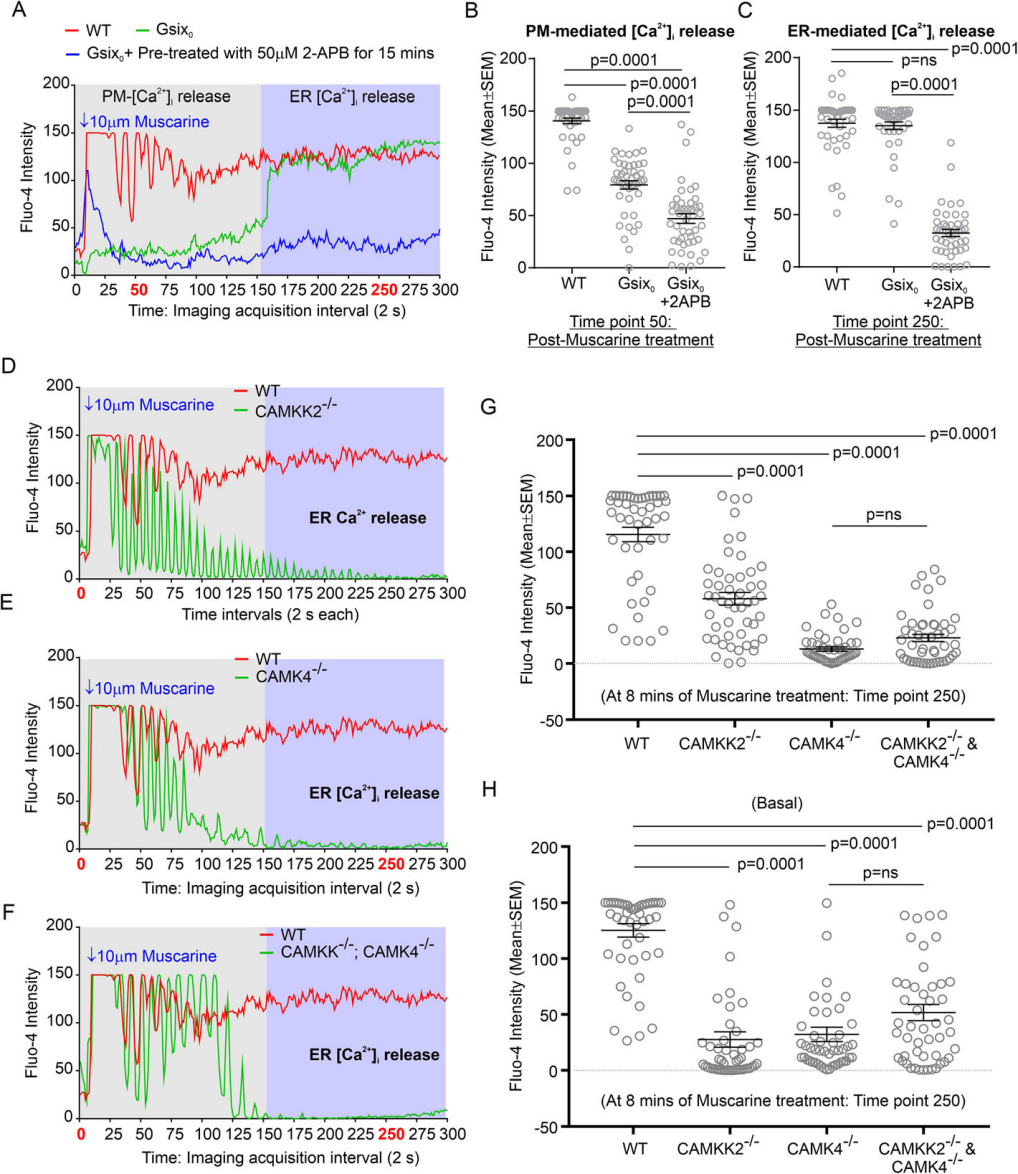
CAMKK2-CAMK4 signaling regulates signal transduction-mediated Ca^2+^ release from the ER. **a**, **d**-**f**: Line graphs showing temporal alterations of Fluo-4 intensity change ([Ca^2+^]_i_) in the wild-type, ΔG_six0_, CAMKK2^−/−^, CAMK4^−/−^, and DKO HEK293 cells treated with 10 μM muscarine chloride. The confocal image acquisition parameters and the treatment condition between different experimental sets were kept uniform; therefore, the relative intensity difference was comparable. At the beginning of the time-lapse imaging, the mean Fluo-4 intensity per cell was set at a threshold below 50 pixel intensity (arbitrary unit) to capture the dynamic range of muscarine-induced Ca^2+^ release in different cell types irrespective of the cells basal [Ca^2+^]_i_ level. (**b**-**c** & **g**): Scatter plots showing the mean Fluo-4 intensity. *N* = 40–50 cells analyzed in 5 independent experiments in each category. *P* values by one-way ANOVA followed by Dunnett’s multiple comparison test. H: Scatter plot showing basal [Ca^2+^]_i_. The basal [Ca^2+^]_i_ in the wild-type, CAMKK2^−/−^, CAMK4^−/−^, and DKO HEK293 cells was measured in a different experiment where no initial threshold was set as mentioned previously, however, the microscopic image capture settings were kept uniform. N = 40–50 cells analyzed in 5 independent experiments in each category. *P* values by one-way ANOVA followed by Dunnett’s multiple comparison test. The entire dataset is presented in Supplementary Fig. S7

The cytosolic free [Ca^2+^]_i_ may not be the limiting factor for receptor-mediated TF internalization [63], but it is essential for vesicular transport along the ER/Golgi pathway [64]. To study the effect of CAMKK2 and/or CAMK4 loss on intracellular [Ca^2+^]_i_, muscarinic signal transduction-mediated Ca^2+^ release response was first characterized using wild-type and G proteins-deficient HEK293 cells. Deletion of six Gα proteins (GαS/L/Q/11/12/13) caused a zero functional G condition (G_Δsix0_) [38]. Treatment of the serum-starved HEK293 cells with 10 μM muscarine caused an immediate (within seconds to a minute) and a delayed (up to 5 mins of observation) [Ca^2+^]_i_ release response (Fig. 7a, red). Treatment of the G_Δsix0_ cells with 10 μM muscarine significantly decreased immediate [Ca^2+^]_i_ release but retained delayed increase of [Ca^2+^]_i_ as observed in the wild-type cells (Fig. 7a-c, green). Further, 10 μM muscarine treatment-induced delayed [Ca^2+^]_i_ release in G_Δsix0_ cells which was significantly decreased following 15 mins pre-treatment of cells with 50 μM 2-amino-ethoxy-diphenylborane (**2-APB**), an inhibitor of inositol 1,4,5-trisphosphate (IP_3_)-receptor (Fig. 7a&c, blue). The 2-APB is a membrane-permeable IP_3_ antagonist [48] which stimulates store-operated calcium (SOC) release at low concentrations (< 10 μM) and inhibits it at higher concentrations (up to 50 μM) [49]. Based on these observations, mAChR agonist (muscarine) induced delayed (~ 3–5 min) [Ca^2+^]_i_ release response was considered as ER-mediated-[Ca^2+^]_i_ release and the immediate response (seconds to a minute) was considered as plasma membrane (**PM**)-mediated [Ca^2+^]_i_ release (Fig. 7a, grey and magenta-colored area respecitvely). Treatment of CAMKK2^−/−,^ CAMK4^−/−^ and DKO HEK293 cells with 10 μM muscarine elicited an immediate PM-mediated [Ca^2+^]_i_ release but failed to cause ER-mediated [Ca^2+^]_i_ release (Fig. 7d-f). In addition, the oscillating nature of the Fluo-4 intensity change within seconds to a minute of muscarine treatment indicates an alternative increase and decrease in intracellular [Ca^2+^]_i_ which may be due to successive opening and closing of the PM-bound non-selective ion channels. The frequency of this response was relatively different between wild-type and CAMKK2 and/or CAMK4-deficient cells which indicates a potential alteration in the PM-bound ion-channels. The ligand-mAChR signaling-mediated ER [Ca^2+^]_i_ release was significantly decreased in CAMKK2^−/−,^ CAMK4^−/−^ and DKO HEK293 cells compared to the wild-type cells (Fig. 7g). In a separate experiment, the basal [Ca^2+^]_i_ level was found significantly low in the CAMKK2^−/−,^ CAMK4^−/−^ and DKO HEK293 cells compared to the wild-type cells (Fig. 7h). Overall, this study indicates that CAMKK2-CAMK4 signaling regulates intracellular Ca^2+^ homeostasis.

### Loss of CAMKK2 and CAMK4 caused aberrant intracellular Ca^2+^ release following treatment with TF

**Fig. 8:**
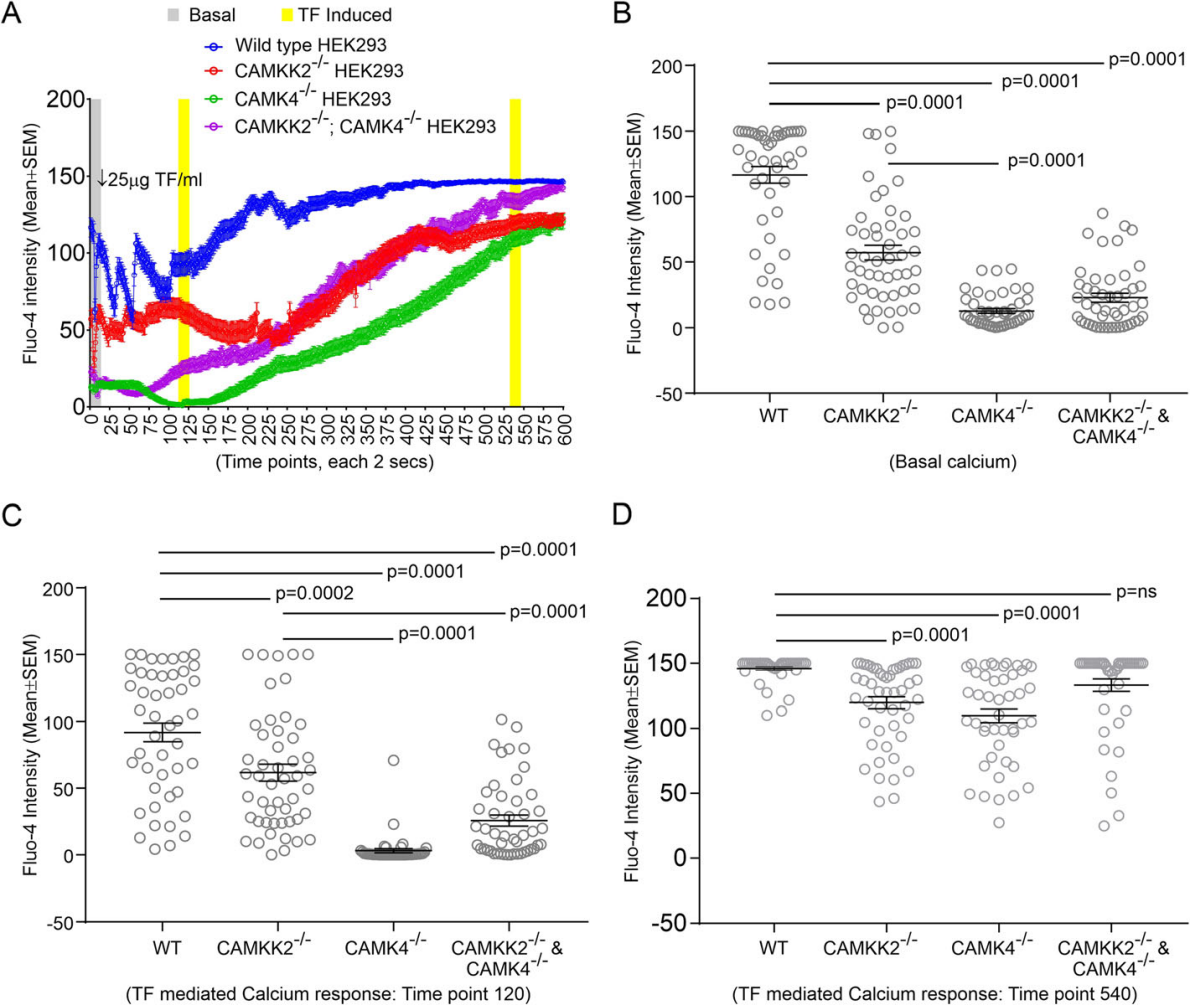
TF-signaling mediated intracellular Ca^2+^ release in CAMKK2^−/−^, CAMK4^−/−^, and DKO HEK293 cells. **a**: Line graphs showing temporal alterations of Fluo-4 intensity change ([Ca^2+^]_i_) in the wild-type, CAMKK2^−/−^, CAMK4^−/−^, and DKO HEK293 cells following treatment with 25 μM TF. The confocal image acquisition parameters and the treatment condition between different experimental sets were kept uniform and therefore, difference in the relative intensity was comparable. **b**: Scatter plot showing basal [Ca^2+^]_i_. N = 40–50 cells analyzed in 5 independent experiments in each category. *P* values by one-way ANOVA followed by Dunnett’s multiple comparison test. The entire dataset is presented in Fig. S7. **c**-**d**: Scatter plots showing the mean Fluo-4 intensity after 2 mins (**c**) and 9 mins (**d**) of TF treatment. N = 40–50 cells analyzed in 5 independent experiments in each category. The entire dataset is presented in Figure S7. *P* values by one-way ANOVA followed by Dunnett’s multiple comparison test

TFRC functions as a signal-transduction molecule for its own recycling via increases in the internal Ca^2+^ concentration [65]. Therefore, TF-induced intracellular calcium homeostasis was measured by Fluo-4 intensity change. The basal [Ca^2+^]_i_ level was found significantly low in CAMKK2^−/−,^ CAMK4^−/−^ and DKO HEK293 cells compared to the wild-type cells indicating reproducibility of the Fluo-4 intensity-based [Ca^2+^]_i_ measurement procedure as shown previously (Fig. 8a-b). Treatment of serum-starved wild-type cells with 25 μg/ml TF caused a [Ca^2+^]_i_ release response which exponentially increased and reached a plateau at ~ 5 mins and subsequently maintained a steady level up to 10 mins of observation (Fig. 8a). Interestingly, the CAMKK2^−/−^, CAMK4^−/−^ and DKO HEK293 cells exhibited significantly altered [Ca^2+^]_i_ release response at ~ 2 and ~ 9 mins of treatment with TF (Fig. 8a & c-d). Overall kinetics of the [Ca^2+^]_i_ release upon TF treatment (Fig. 8A) indicated that loss of CAMKK2 and /or CAMK4 significantly decreased TF-TFRC signaling-mediated intracellular [Ca^2+^]_i_ release (Fig. 8c-d). Together, these results indicate that loss of CAMKK2 or CAMK4 function disturbed intracellular [Ca^2+^]_i_ homeostasis during TF uptake which may be responsible for abnormal TF trafficking.

## Discussion

This study provided evidence that loss of Camk4 leads to abnormal TF turnover and iron dyshomeostasis in different mouse tissues which supported the hypothesis that abnormal Ca^2+^ signaling and iron dyshomeostasis are linked through CAMKK2-CAMK4 signaling-mediated regulation of TF trafficking. The cholinergic signaling-based study using G_six0_, CAMKK2^−/−^, CAMK4^−/−^ and DKO HEK293 cells provided evidence that CAMKK2-CAMK4 signaling regulates ER-mediated Ca^2+^ homeostasis which was linked to abnormal calcium signaling during TF trafficking, interaction of TF/TFRC associated MPCs, and altered PTMs of TF/TFRC. Overall, this study provided a novel mechanistic link between TF trafficking, Ca^2+^ signaling and cellular iron homeostasis, all regulated by CAMKK2-CAMK4 signaling.

The Camk4^−/−^ mouse tissue-based study indicated that loss of Camk4 significantly increased total Tf/Tfrc content in the cerebellum but decreased it in the liver compared to the wild-type, which is in agreement with similar observation in the corresponding tissues in a previous study using the Camkk2^−/−^ mouse [9]. It is important to note that in the Camk4^−/−^ mice, the iron content was significantly increased in both cerebellum and liver tissues, whereas the Tf/Tfrc content was increased in the cerebellum but decreased in the liver compared to the wild-type. Liver secretes Tf to harvest iron from the gut and supply Tf-bound iron to different organs. The highest level of Tf mRNA was detected in the liver and a lower level was observed in the cerebellum and other organs in rodents [66–68]. Therefore, it is tempting to suggest that the whole-body Camk4 deficiency caused a unidirectional loading of Tf-bound iron to the brain through systemic circulation. Alternatively, transcriptional regulation of Tf and Tfrc by Camk4 cannot be excluded. Tf expression is regulated by the binding of cAMP-responsive element-binding proteins (**CREB**) to the promoter of Tf in oligodendrocytes [69]. Similar regulation of Tf and Tfrc gene expression by Creb has been reported in Sertoli [70] and erythroid cells [71] respectively. Camk4 phosphorylates transcriptional activator Creb1 on ‘Ser-133’ and controls neuronal gene expression and memory function [34, 72]. Thus, it is possible that loss of Camk4-Creb signaling may affect the expression of Tf and Tfrc in a tissue-specific manner. Interestingly, Camk4 is expressed in the cortex, but its loss exhibited no effect on Tf and iron content in the cortex (within detection limit), which suggests that other factors may be involved. Furthermore, the significant reduction of a negatively charged fraction of Tf (pH ~ 3–4) in Camk4^−/−^ cerebellum and liver tissues is in agreement with similar findings in a previous study using Camkk2^−/−^ mice [9]. The pH ~ 3–4 fraction of Tf contains multiple Ser/Thr and Tyr phosphorylated residues at functionally relevant sites [9]. The significant decrease in the pH ~ 3–4 fraction of Tf in Camk4-decificent tissues indicates that Camkk2-Camk4 signaling regulates phosphorylation of Tf in vivo. Also, the charged fractions of Tf and its high HMW forms showed a tissue-specific difference in Camk4 loss-of-function condition, which indicates a complex pattern of PTMs, possibly regulated by multiple mediators. Overall, this study indicates that Camk4 controls tissue-specific turnover and PTMs of both Tf and Tfrc which is associated with iron dyshomeostasis.

The defective muscarine (**agonist**) treatment-induced Ca^2+^ release response in CAMKK2/CAMK4-deficient cells indicated a role of CAMKK2-CAMK4 signaling in the maintenance of intracellular Ca^2+^ homeostasis. The purpose of using G_Δsix0_ HEK293 cells (zero functional G) and IP_3_R antagonist (2-APB) in muscarinic signal-transduction-mediated Ca^2+^ release study was to delineate the temporal alteration in ER-mediated Ca^2+^ release, which was then used to characterize the effect of CAMKK2 and/or CAMK4 on ER-mediated Ca^2+^ homeostasis during TF trafficking. Within seconds to minutes of muscarine binding to mAChRs, trimeric G proteins associate with effector proteins within a signaling cascade and activate phospholipase C, generates IP_3_ and cAMP, influence Ca^2+^ entry across the plasma membrane, and cause release of Ca^2+^ from the intracellular stores [73–75]. The second wave of signaling is dependent on the recruitment of β-arrestins and subsequent desensitization followed by internalization of the receptor which extends for minutes to hours after agonist binding [76]. Modulation of ion channels by mAChR agonists is very complex and context-dependent, for example, both K^+^ and voltage-gated Ca^2+^ channels are either activated or inhibited by mAChR signaling which depends on the cell-type [77]. The voltage-gated Ca^2+^ channels are key mediators of depolarization-induced Ca^2+^ influx. The G protein βγ dimer (G_βγ_) regulates several plasma membrane-bound Ca^2+^ channels [78, 79]. The IP_3,_ generated in the first wave of mAChRs signaling, is soluble and diffuses through the cell, where it binds to its receptor - IP_3_R, which is a ligand-gated Ca^2+^ channel located in the ER [80]. When IP_3_ binds IP_3_R, [Ca^2+^]_i_ is released into the cytosol, thereby activating various Ca^2+^-regulated intracellular signals [80]. The increase in [Ca^2+^]_i_ observed following muscarinic activation of HEK293 cells was as expected and it agreed with another study using acetylcholine as a native mAChR agonist [81]. It is important to note that there is considerable heterogeneity in the agonist-induced [Ca^2+^]_i_ in HEK293 cell populations (Fig. S7). Similar heterogeneity in the Fluo-4-based confocal imaging of cytosolic Ca^2+^ for Gα_i_-coupled GPCR-signaling has been reported in HeLa cells [82]. Such heterogeneity may be due to clonal variations in the transformed cells. The zero functional G condition eliminated the immediate Ca^2+^ release response (within a minute) but retained the delayed (~ 2–5 min) response (Fig. 7). Previously, in a similar experiment, it was shown that replacement of CaCl_2_ with BaCl_2_ in the extracellular fluid significantly reduced the immediate Ca^2+^ release during muscarinic activation of HEK293 cells, which indicates potential involvement of PM-operated voltage-gated Ca^2+^ channels [9]. The delayed response was completely abolished following IP_3_R antagonist 2-APB treatment in G_Δsix0_ cells (Fig. 7). Based on this, the immediate response was considered PM-mediated and the delayed response was considered as ER-mediated Ca^2+^ release. The exact mechanism of ER-mediated Ca^2+^ release in zero functional G condition is not known. The delayed nature of this response may suggest potential involvement of β-arrestin. Recently, β-arrestin-1-mediated, G protein-independent, stimulation of [Ca^2+^]_i_ flux has been reported by signal transduction between GPCR and transient receptor potential (**TRP**) cation channel subfamily C3 (**TRPC3**) channel [83]. The TRP ion channels are a superfamily of Ca^2+^-permeable cation nonselective channels present on both PM and intracellular membranes [84]. In addition, 2-APB is known to block TRP channels [85, 86]. Therefore, biased arrestin signaling in muscarine stimulated G_Δsix0_ HEK293 cells may be responsible for the delayed ER-mediated Ca^2+^ release response.

This study provided evidence that receptor-mediated TF trafficking elicited a rise in the [Ca^2+^]_i_ in HEK293 cells which was significantly decreased in CAMKK2 and/or CAMK4-deficient cells compared to the wild-type. In the light of previous discussion, where role of CAMKK2/CAMK4 signaling in the regulation of intracellular Ca^2+^ homeostasis has been delineated, it is rational to suggest that there is a connection between the disturbance in intracellular Ca^2+^ homeostasis and defective TF-trafficking in CAMKK2/CAMK4-deficient cells. The role of Ca^2+^ in several constitutive vesicular transport processes, including endosome fusion, exosome release and cargo transport from the ER to Golgi has been well documented [64, 87, 88]. It has been shown that the endosomal recycling of radiolabeled TF is dependent on intracellular [Ca^2+^]_i_ [89]. Also, Ca^2+^ chelators, Ca^2+^-channel inhibitor, and CAM-antagonist were shown to drastically reduce TFRC recycling [65]. Further, artificial elevation of intracellular [Ca^2+^]_I_ accelerated TF recycling rate [65]. Thus, the significantly reduced basal [Ca^2+^]_i_ and the defect in ER-mediated Ca^2+^ release during signal transduction in CAMKK2 and/or CAMK4-deficient HEK293 cell clones indicates a potential link between impaired intracellular Ca^2+^ homeostasis and abnormal TF trafficking.

The BN-PAGE study provided a unique in-depth view of the alterations in TF/TFRC/CAMKK2/CAMK4 associated MPCs which is regulated by CAMKK2-CAMK4 signaling. The first-dimension BN-PAGE separated native proteins in non-denatured and non-reducing conditions, whereas the second-dimension SDS-PAGE separation was under reduced and denatured conditions [40]. The association of CAMKK2, TFRC, and TF in the same MPCs (> 1200 kDa) and its dramatic reduction during receptor-mediated TF trafficking in CAMKK2 and/or CAMK4-deficient cells indicates a functional dependency between these protein complexes (Fig. 4). Also, the alterations in CAMKK2, CAMK4, and endogenous TF-associated ~ 146 kDa MPCs in CAMKK2 and/or CAMK4-deficient cells, and the absence of TFRC in these MPCs suggest additional control of TF trafficking by CAMKK2-CAMK4 signaling which is independent of binding to the TFRC. The molecular weight of TF and TFRC is 77 and 84 kDa respectively. While the majority of the native TF, TFRC, and RFP-TFRC appeared as a p75 (TF), p100 (TFRC) and p135kDa (RFP-TFRC) proteins respectively, a smaller fraction of each protein appeared as high molecular weight forms, particularly in the BN-PAGE and IEF-based studies. The HMW forms of TFRC and TF may not be due to the dimerization of the proteins. Both TF and TFRC form dimers by intermolecular disulfide bonds involving cysteine residues [90, 91]. The disulfide bridges between the sulfhydryl groups on cysteines are usually separated by chemical reduction of the disulfide bonds using DTT [92]. The use of 100-150 mM DTT in the second dimension SDS-PAGE ensured reduction of all disulfide linkages. Thus, dimerization can be excluded as a possible explanation for the appearance of HMW forms. Another possibility is PTMs that may add considerable molecular mass, for example mono-ubiquitination and mono-sumoylation can add approximately 8–10 kDa mass. Interestingly, The PhosphositePlus database [54] archived ubiquitination at multiple lysine residues on both TF [93] and TFRC [94–96]. Detailed mass spectrometric characterization is required in the future to identify the nature of the PTMs responsible for additional mass gain in these proteins. The HMW forms of TFRC also exhibited a dramatic difference in charge shift (pI) during TF trafficking as revealed by the IEF-based study in CAMKK2/CAMK4-deficient cells which indicates some involvement of these kinases in mediating the PTMs. Interestingly, phosphorylation of TFRC at multiple residues, for example P-S19/Y20/T21/S24/S34/Y282/T286 [97, 98], has been documented in Phosphositeplus database. In an endothelial cell-based TF trafficking study (Revised manuscript submitted in BBA-Molecular Cell Research, in review), treatment of intracellular p80 and p100kDa forms of TF with a cocktail of deglycosylase to remove all N and O-linked glycans, resulted in a shift of ~ 5 kDa mass. This indicates that glycosylation may not be the only PTMs responsible for the HMW forms. Recently, there has been a renaissance of interest in the ability of protein modifications including ubiquitin or sumo-like proteins to moieties such as sugars, prenyl groups and covalent attachment of lipids to the target-specific sites in proteins to coordinately exert control over protein function in diverse cell biological contexts. In addition, ER serves as a hub for post-translation modifications of proteins [99], therefore, altered Ca^2+^ homeostasis in the ER in CAMKK2 and or CAMK4-deficient cells may affect PTMs of TF. Therefore, based on the above discussion, it is tempting to suggest that CAMKK2-CAMK4 signaling may control intracellular repertoire of receptor-bound TF trafficking through a complex array of PTMs by recruiting effector proteins that are functionally dependent on ER Ca^2+^ homeostasis.

## Conclusions

This study provided a link between intracellular Ca^2+^ and iron homeostasis, both regulated by CAMKK2-CAMK4 signaling which is vital for cell function and survival. The findings may provide an explanation for the behavioral abnormalities observed in both Camkk2^−/−^ and Camk4^−/−^ mice [26, 100]. In particular, the locomotor defects associated with the altered cerebellar function in Camk4^−/−^ mice [100] correlated with an increased iron deposition in the cerebellum as reported in this study. The increased iron deposition and disturbed Ca^2+^ homeostasis in the Camk4-deficient cerebellar neurons may provide an explanation for the behavioral abnormalities due to disruption of neuronal function. Cerebellum is a critical node in the distributed neural circuits sub-serving not only motor function but also autonomic, limbic and cognitive behaviors [101]. Emerging evidence suggest cerebellar contribution to the cognitive and neuropsychiatric deficits in neurodegenerative diseases, specifically AD [101–103]. Amyloid plaques have been observed in the cerebellum of AD patients [104–107]. Therefore, future studies must prioritize CAMKK2-CAMK4 signaling mediated TF trafficking in different parts of the brain to understand iron-overloading mediated neurodegeneration and cognitive malfunction.

## Supplementary information

**Additional file 1: Figure-S1:** Generation of CRISPR/Cas9-mediated CaMKK2^−/−^, CaMK4^−/−^, and DKO HEK293 cell clones. (A-C): Fluorescence-activated sorting of Cas9-reporter (GFP) expressing cells. The GFP positive cells were plated at a single cell density in 96 well plates and subsequently CAMK4 and CAMKK2 expression was examined by immunoblotting (D-G) to identify CAMKK2^**−/−**^, CAMK4^**−/−**^ and DKO clones. CAMKK2 clone A5 [9] was used for DKO generation. (D-G): Immunoblots showing expression of CAMK4, and GAPDH in multiple CAMK4^**−/−**^ and DKO HEK293 cell clones. (H): Immunoblots showing loss of expression of CAMK4 and CAMKK2 in DKO HEK293 cell clone-D6. (I): Diagrammatic representation of the two-dimensional BN-PAGE/SDS-PAGE analysis.

**Additional file 2: Figure-S2:** Characterization of alternatively spliced isoforms of Camkk2 in mouse tissues. (A): Genomic organization of Camkk2 showing two major isoforms. Black rectangles represent exons. Some exons are grey colored to distinguish it from the closely spaced exons. The genomic locations and the sequence of the primers used to amplify the ORF encompassing exon 16 are marked by arrows. Colored segment of the F2 primer indicates the sequence from adjacent exons (B): Clustal Omega Sequence alignment [109] showing the protein sequences of CAMKK2 isoforms. Swiss-Prot manually annotated and reviewed sequences from *Homo sapiens* (Human) and *Mus musculus* (Mouse) was presented. An asterisk indicates positions which have a single, fully conserved residue. A colon indicates conservation between groups of strongly similar properties. A period indicates conservation between groups of weakly similar properties. The bold red-colored residue overlaps splice site. Exons are alternatively colored black, blue and red. The bold small residues are PTMs listed in the PhosphositePlus database. (C-D): Agarose gel showing amplification of the Camkk2^+ 16^ and Camkk2^Δ16^-specific PCR products. (E-F): Agarose gel showing amplified Camkk2-isoforms in mouse liver tissue (E) and subsequent gel-excision-based purified PCR products (F). (G-H): Chromatograms showing DNA sequences of ~ 300 (top band) and ~ 200 (bottom band) bp amplicons.

**Additional file 3.Figure-S3:** BLAT alignment of the ~ 300 bp amplicon-derived DNA sequence corresponding to Camkk2^Δ16^ isoform. (A-D): BLAT alignments showing the exon structure of Camkk2 isoforms and alignment of the ~ 300 bp amplicon-derived sequence. The exons are color-coded. (E): Nucleotide sequence and the corresponding amino acid sequence representing a partial reading frame of Camkk2^+ 16^ isoform. (F): Translational of ~ 300 bp amplicon-derived DNA sequence. The colored sections represent the exons matched to Camkk2^+ 16^ isoform. Note the absence of Camkk2 exon 16 (cyan highlighted). The non-highlighted segments represent additional sequence gain which is not documented in the mouse genome (GRCm38/mm10) assembly. This may be due to strain-specific variation.

**Additional file 4: Figure-S4**: BLAT alignment of the ~ 200 bp amplicon-derived DNA sequence corresponding to Camkk2^+ 16^ isoform. (A): BLAT alignments showing the exon structure of Camkk2 isoforms and alignment of the ~ 200 bp amplicon-derived sequence. The exons are color-coded. (B-C): Nucleotide sequence and the corresponding amino acid sequence representing the ~ 200 bp amplicon-derived DNA sequence (B) and a partial reading frame of Camkk2^+ 16^ isoform (C) showing identical match.

**Additional file 5: Figure-S5:** Relative amount of TF and TFRC inCamk4^−/−^ mouse cortex tissues. A-B: Immunoblot showing relative amount of TF and TFRC in cortex tissues. A p50 anti-TF positive band was found dramatically reduced in Camk4^−/−^ mice cortex tissues compared to the wild-type. The p50 band may be due to proteolysis of TF which needs to be validated by mass spectrometry in the future. The bottom panel represents Oriole-stained total protein loading. The red arrow indicates the band used for quantifying TF and TFRC. C-D: Scatter plots showing relative abundance of Tf and Tfrc in the cortex tissues. *N* = 2 replicates from three wild-type and Camk4^−/−^ mice. *P* values by t-test (unpaired).

**Additional file 6: Figure-S6.** Co-migration of constitutively expressed native TF and TFRC-associated MPCs during trafficking in HEK293 cells**.** (A): Immunoblots showing increased constitutive expression of TFRC in HEK293 cells grown in OPti-MEM + 5%FBS media compared to DMEM+ 10% media at different time points. The cells were grown in DMEM media for 72 h. Note the presence of p120 TFRC at 72 h of expression. (B-C): Alterations of TFRC-associated MPCs in TF-treated (25 μg/ml for 30 mins) and untreated HEK293 cells grown in Opti-MEM + 5%FBS media for 72 h. The MPCs in different treatment conditions were separated together in the same first-dimension BN-PAGE; therefore, their relative migration is comparable. The separation of Coomassie-stained native page markers is provided at the top of the immunoblots (B-D). The immunoblots are aligned vertically to show the relative migration of the protein complexes. Red and green square, as well as arrows, indicate the relative shift of ~ 480 kDa TFRC-associated MPCs following TF-treatment compared to untreated cells. (D): The anti-TFRC immunoblots from B and C were false-colored and overlaid to show co-migration of the TFRC-associated MPCs during trafficking in the TF-treated vs untreated HEK293 cells. (E-F): The immunoblots presented in C and D are incubated with anti-TF antibody and visualized. Red and green rectangles are indicating a relative shift in co-migrated TF and TFRC associated protein complexes following TF-treatment compared to untreated cells. (G): The immunoblots presented in E and F are false-colored and overlaid to show co-migration and vertical alignment of TF and TFRC associated protein complexes. White arrow indicates that TF-treatment shifted both complexes to a relatively higher molecular weight region.

**Additional file 7: Figure-S7:** Muscarinic signal transduction-mediated calcium release response in ΔG_six0,_ CaMKK2^−/−^, CaMK4^−/−^, and DKO HEK293 cell clones. (A-D): Line graphs showing the alterations of Fluo-4 intensity ([Ca^2+^]_i_) in the wild-type, ΔG_six0_, CAMKK2^−/−^, CAMK4^−/−^, and DKO HEK293 cells following 10 μM Muscarine chloride treatment (A-D) or 25 μM TF (E-I) treatment.

## Abbreviations

DKO: Double knockout
BN-PAGE/SDS-PAGE: Blue native polyacrylamide gel electrophoresis/ sodium dodecyl sulphate polyacrylamide gel electrophoresis
IEF: Isoelectric focusing
