# Supplementary figures and images for: CAMKK2-CAMK4 signaling regulates transferrin trafficking, turnover, and iron homeostasis
Source: Cell Commun Signal. 2020 May 27;18:80. doi: 10.1186/s12964-020-00575-0 (PMC7251913; doi:10.1186/s12964-020-00575-0)

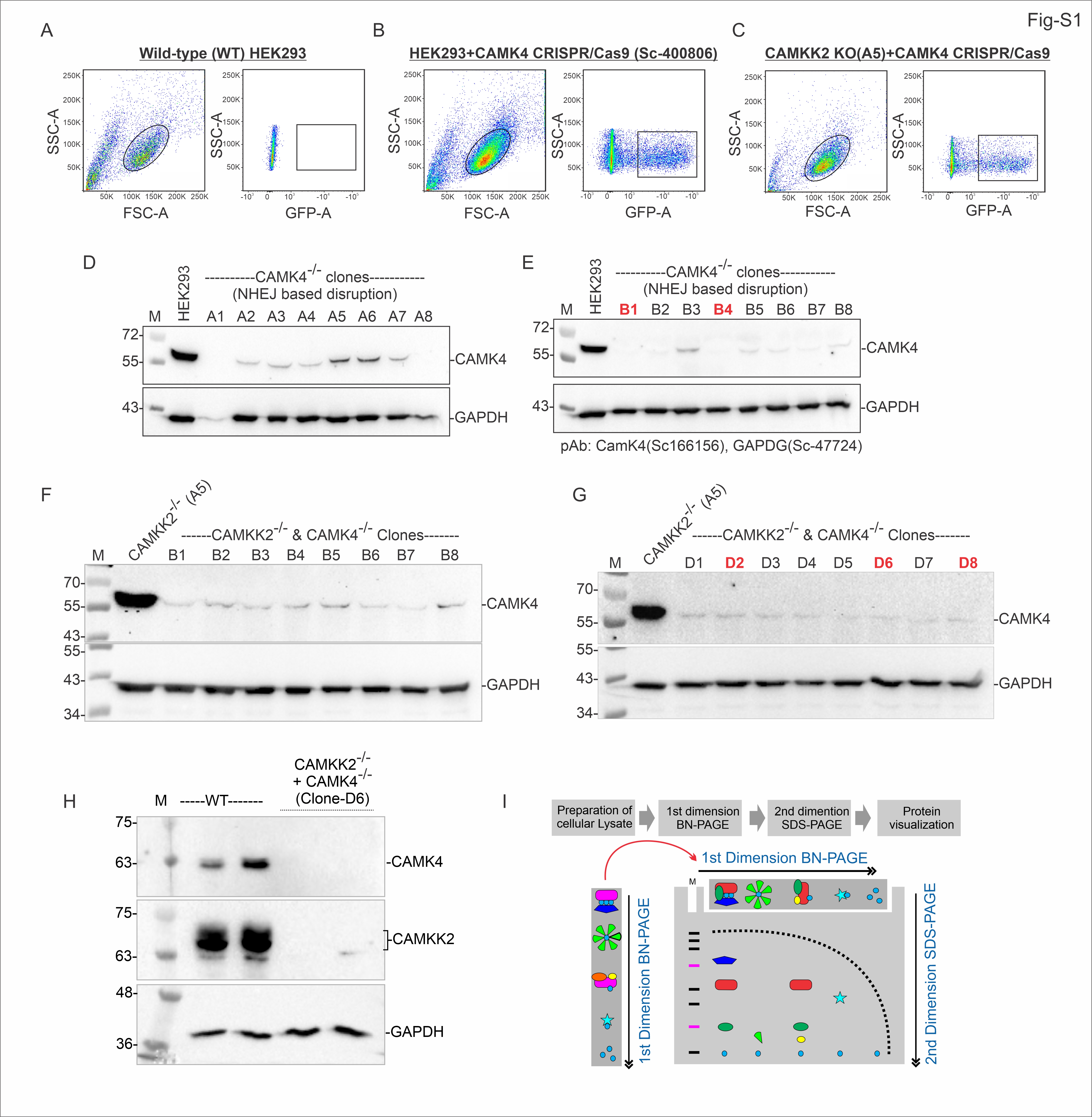

Supplement: Supplementary file 2 — Additional file 1: Figure-S1: Generation of CRISPR/Cas9-mediated CaMKK2−/−, CaMK4−/−, and DKO HEK293 cell clones. (A-C): Fluorescence-activated sorting of Cas9-reporter (GFP) expressing cells. The GFP positive cells were plated at a single cell density in 96 well plates and subsequently CAMK4 and CAMKK2 expression was examined by immunoblotting (D-G) to identify CAMKK2−/−, CAMK4−/− and DKO clones. CAMKK2 clone A5 [9] was used for DKO generation. (D-G): Immunoblots showing expression of CAMK4, and GAPDH in multiple CAMK4−/− and DKO HEK293 cell clones. (H): Immunoblots showing loss of expression of CAMK4 and CAMKK2 in DKO HEK293 cell clone-D6. (I): Diagrammatic representation of the two-dimensional BN-PAGE/SDS-PAGE analysis. [file 12964_2020_575_MOESM2_ESM.jpg]

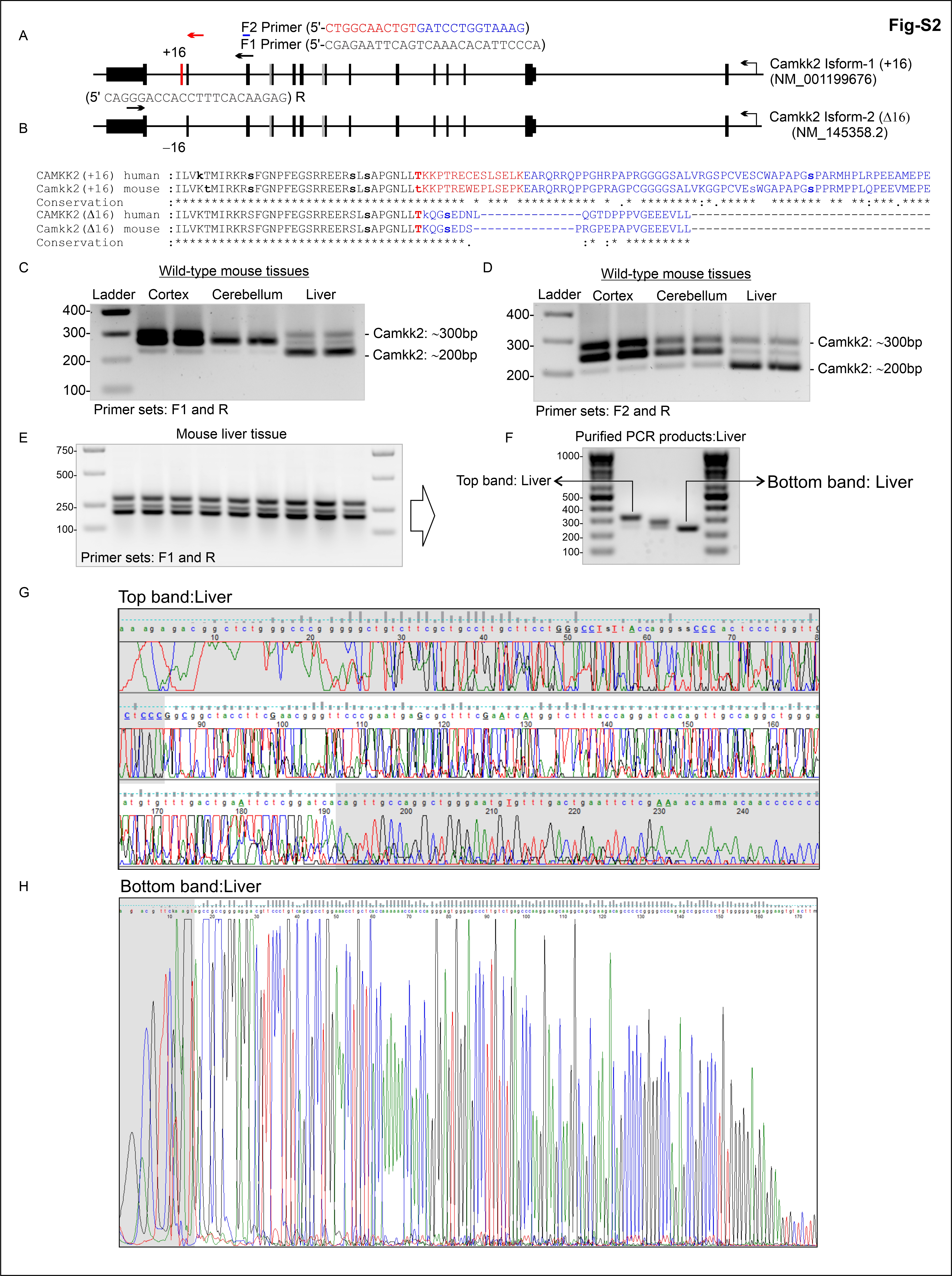

Supplement: Supplementary file 3 — Additional file 2: Figure-S2: Characterization of alternatively spliced isoforms of Camkk2 in mouse tissues. (A): Genomic organization of Camkk2 showing two major isoforms. Black rectangles represent exons. Some exons are grey colored to distinguish it from the closely spaced exons. The genomic locations and the sequence of the primers used to amplify the ORF encompassing exon 16 are marked by arrows. Colored segment of the F2 primer indicates the sequence from adjacent exons (B): Clustal Omega Sequence alignment [109] showing the protein sequences of CAMKK2 isoforms. Swiss-Prot manually annotated and reviewed sequences from Homo sapiens (Human) and Mus musculus (Mouse) was presented. An asterisk indicates positions which have a single, fully conserved residue. A colon indicates conservation between groups of strongly similar properties. A period indicates conservation between groups of weakly similar properties. The bold red-colored residue overlaps splice site. Exons are alternatively colored black, blue and red. The bold small residues are PTMs listed in the PhosphositePlus database. (C-D): Agarose gel showing amplification of the Camkk2+ 16 and Camkk2Δ16-specific PCR products. (E-F): Agarose gel showing amplified Camkk2-isoforms in mouse liver tissue (E) and subsequent gel-excision-based purified PCR products (F). (G-H): Chromatograms showing DNA sequences of ~ 300 (top band) and ~ 200 (bottom band) bp amplicons. [file 12964_2020_575_MOESM3_ESM.jpg]

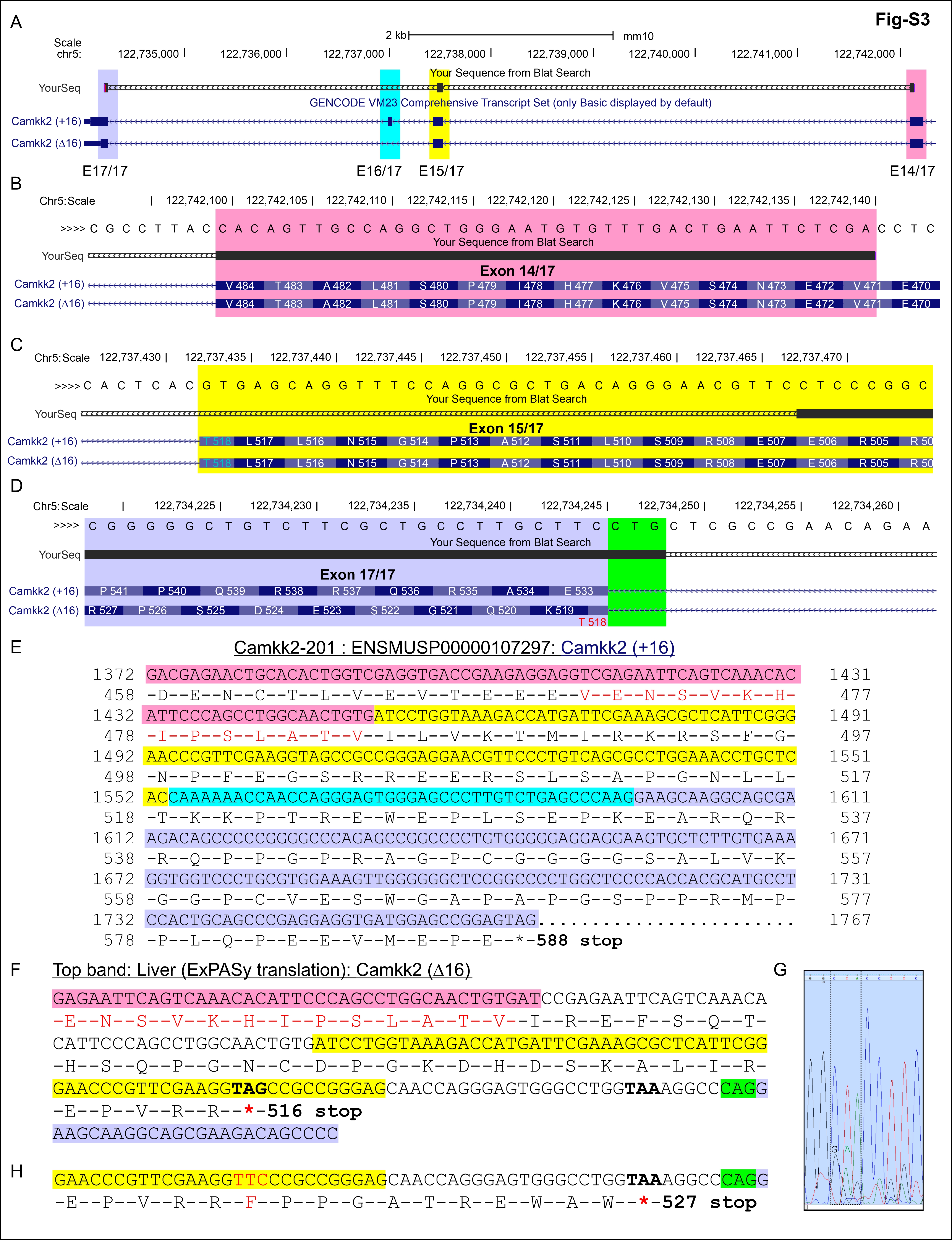

Supplement: Supplementary file 4 — Additional file 3.Figure-S3: BLAT alignment of the ~ 300 bp amplicon-derived DNA sequence corresponding to Camkk2Δ16 isoform. (A-D): BLAT alignments showing the exon structure of Camkk2 isoforms and alignment of the ~ 300 bp amplicon-derived sequence. The exons are color-coded. (E): Nucleotide sequence and the corresponding amino acid sequence representing a partial reading frame of Camkk2+ 16 isoform. (F): Translational of ~ 300 bp amplicon-derived DNA sequence. The colored sections represent the exons matched to Camkk2+ 16 isoform. Note the absence of Camkk2 exon 16 (cyan highlighted). The non-highlighted segments represent additional sequence gain which is not documented in the mouse genome (GRCm38/mm10) assembly. This may be due to strain-specific variation. [file 12964_2020_575_MOESM4_ESM.jpg]

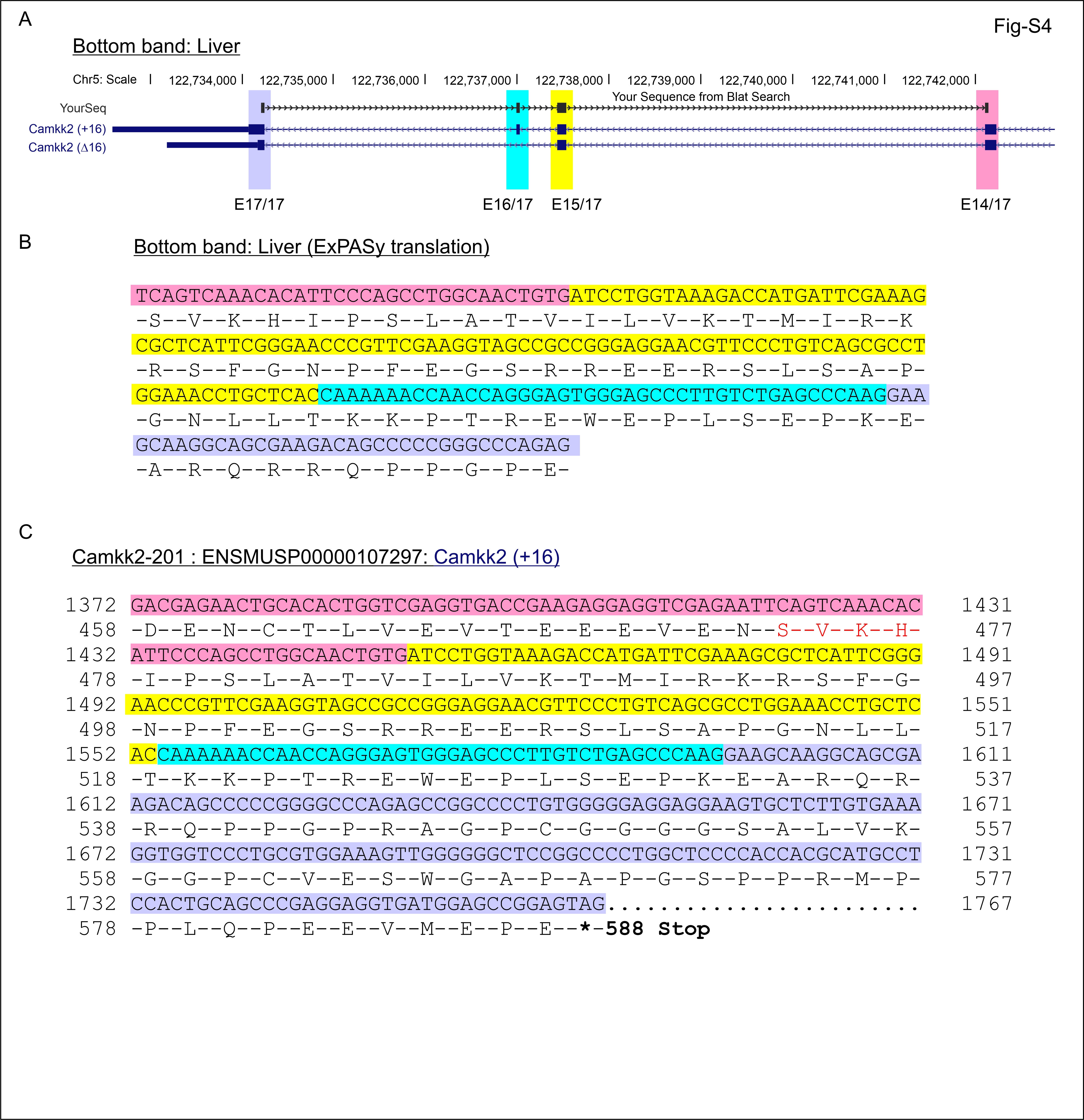

Supplement: Supplementary file 5 — Additional file 4: Figure-S4: BLAT alignment of the ~ 200 bp amplicon-derived DNA sequence corresponding to Camkk2+ 16 isoform. (A): BLAT alignments showing the exon structure of Camkk2 isoforms and alignment of the ~ 200 bp amplicon-derived sequence. The exons are color-coded. (B-C): Nucleotide sequence and the corresponding amino acid sequence representing the ~ 200 bp amplicon-derived DNA sequence (B) and a partial reading frame of Camkk2+ 16 isoform (C) showing identical match. [file 12964_2020_575_MOESM5_ESM.jpg]

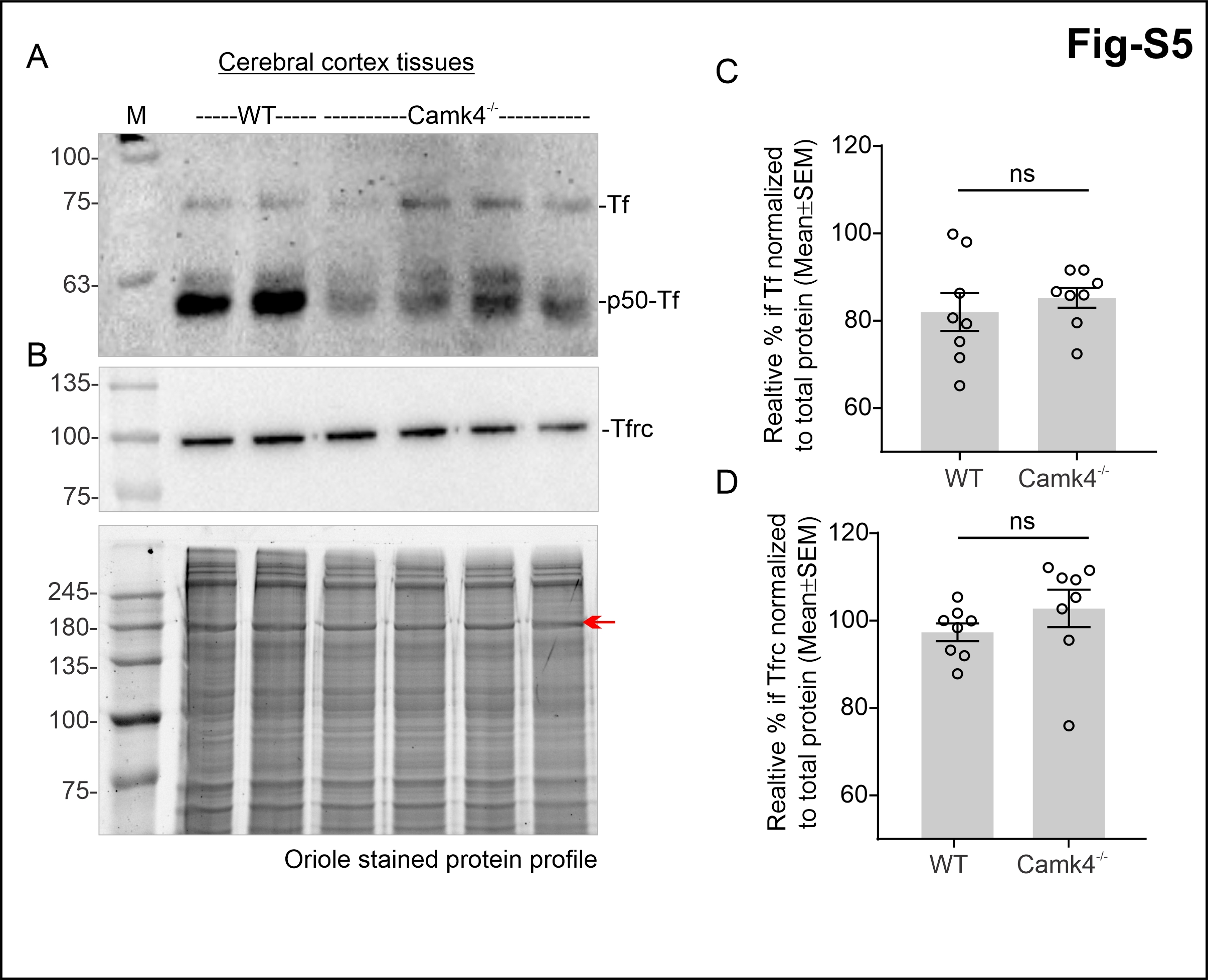

Supplement: Supplementary file 6 — Additional file 5: Figure-S5: Relative amount of TF and TFRC inCamk4−/− mouse cortex tissues. A-B: Immunoblot showing relative amount of TF and TFRC in cortex tissues. A p50 anti-TF positive band was found dramatically reduced in Camk4−/− mice cortex tissues compared to the wild-type. The p50 band may be due to proteolysis of TF which needs to be validated by mass spectrometry in the future. The bottom panel represents Oriole-stained total protein loading. The red arrow indicates the band used for quantifying TF and TFRC. C-D: Scatter plots showing relative abundance of Tf and Tfrc in the cortex tissues. N = 2 replicates from three wild-type and Camk4−/− mice. P values by t-test (unpaired). [file 12964_2020_575_MOESM6_ESM.jpg]

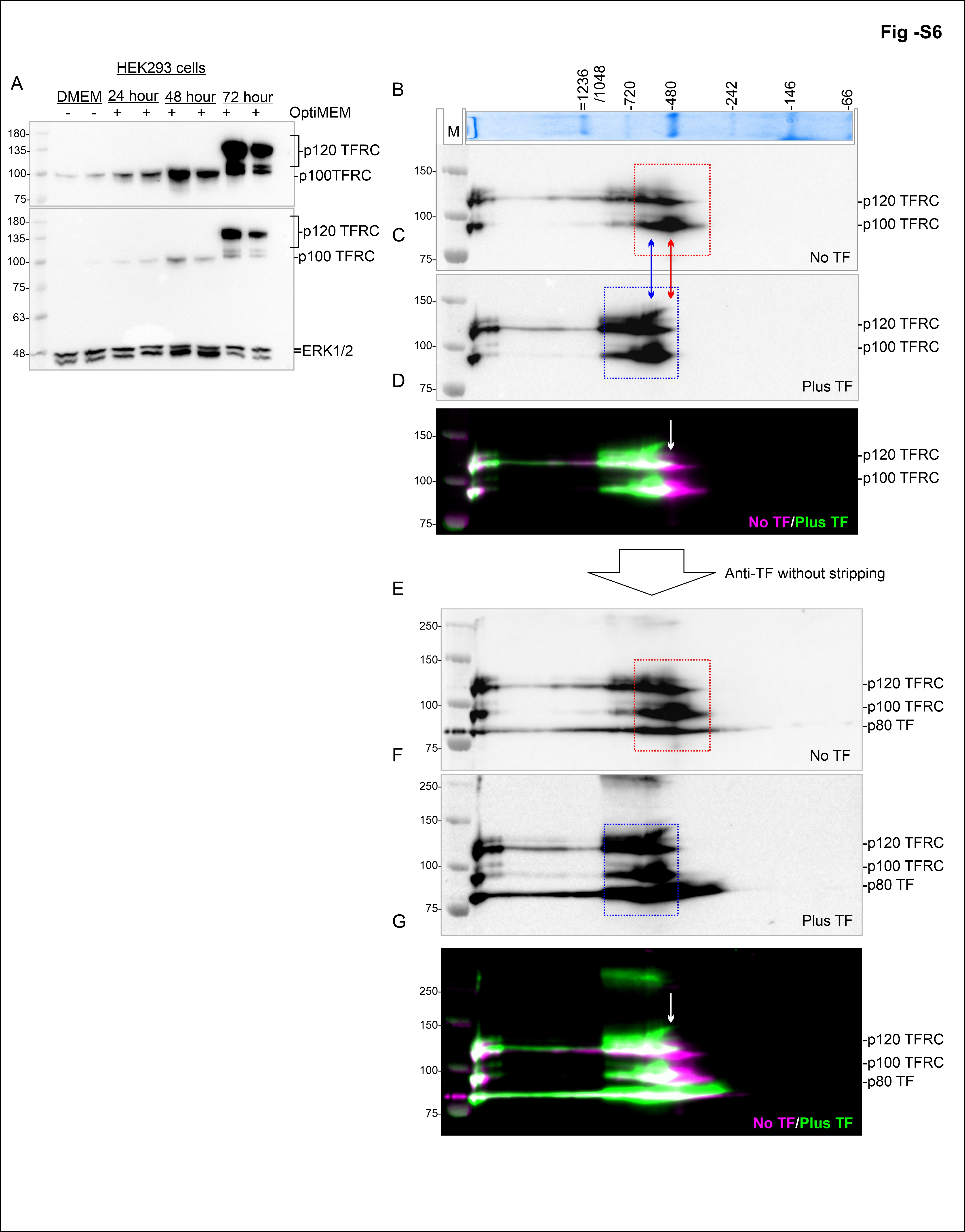

Supplement: Supplementary file 7 — Additional file 6: Figure-S6. Co-migration of constitutively expressed native TF and TFRC-associated MPCs during trafficking in HEK293 cells. (A): Immunoblots showing increased constitutive expression of TFRC in HEK293 cells grown in OPti-MEM + 5%FBS media compared to DMEM+ 10% media at different time points. The cells were grown in DMEM media for 72 h. Note the presence of p120 TFRC at 72 h of expression. (B-C): Alterations of TFRC-associated MPCs in TF-treated (25 μg/ml for 30 mins) and untreated HEK293 cells grown in Opti-MEM + 5%FBS media for 72 h. The MPCs in different treatment conditions were separated together in the same first-dimension BN-PAGE; therefore, their relative migration is comparable. The separation of Coomassie-stained native page markers is provided at the top of the immunoblots (B-D). The immunoblots are aligned vertically to show the relative migration of the protein complexes. Red and green square, as well as arrows, indicate the relative shift of ~ 480 kDa TFRC-associated MPCs following TF-treatment compared to untreated cells. (D): The anti-TFRC immunoblots from B and C were false-colored and overlaid to show co-migration of the TFRC-associated MPCs during trafficking in the TF-treated vs untreated HEK293 cells. (E-F): The immunoblots presented in C and D are incubated with anti-TF antibody and visualized. Red and green rectangles are indicating a relative shift in co-migrated TF and TFRC associated protein complexes following TF-treatment compared to untreated cells. (G): The immunoblots presented in E and F are false-colored and overlaid to show co-migration and vertical alignment of TF and TFRC associated protein complexes. White arrow indicates that TF-treatment shifted both complexes to a relatively higher molecular weight region. [file 12964_2020_575_MOESM7_ESM.jpg]

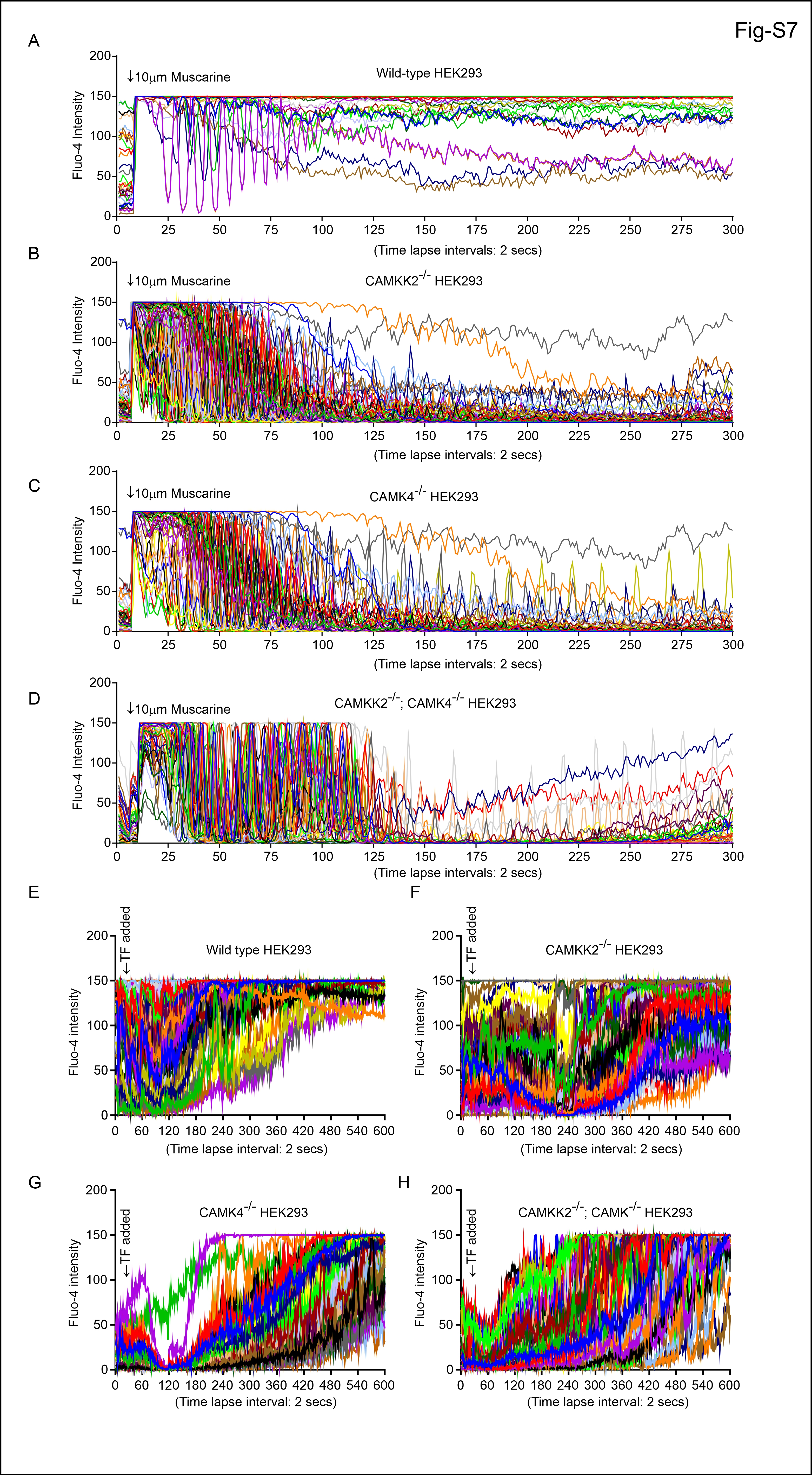

Supplement: Supplementary file 8 — Additional file 7: Figure-S7: Muscarinic signal transduction-mediated calcium release response in ΔGsix0, CaMKK2−/−, CaMK4−/−, and DKO HEK293 cell clones. (A-D): Line graphs showing the alterations of Fluo-4 intensity ([Ca2+]i) in the wild-type, ΔGsix0, CAMKK2−/−, CAMK4−/−, and DKO HEK293 cells following 10 μM Muscarine chloride treatment (A-D) or 25 μM TF (E-I) treatment. [file 12964_2020_575_MOESM8_ESM.jpg]
